# Nanosensitizer for enhanced radiotherapy via tumor microenvironment reshaping and ROS amplification

**DOI:** 10.7150/thno.112311

**Published:** 2026-01-01

**Authors:** Junchao Qian, Yijun Lu, Dandan Wang, Shichuan Zhong, Xiao Liu, Xin Lai, Ziwen Wei, Guangyu Ju, Jinying Yang, Zimeng Wang, Kaiwei Wang, Yue Li, Hongcang Gu, Jian You, Shuanghu Yuan

**Affiliations:** 1 Department of Radiation Oncology, The First Affiliated Hospital of USTC, Division of Life Sciences and Medicine, University of Science and Technology of China, Hefei, Anhui, 230031, P.R. China.; 2 Department of Radiation Oncology, Anhui Provincial Cancer Hospital, Hefei, Anhui, 230031, P.R. China.; 3 Anhui Province Key Laboratory of Medical Physics and Technology, Institute of Health and Medical Technology, Hefei Institutes of Physical Science, Chinese Academy of Sciences, Hefei 230031, P.R. China.; 4 College of Pharmaceutical Sciences, Zhejiang University, 866 Yuhangtang Road, Hangzhou, Zhejiang, 310058, P. R. China.; 5 Key Lab of Materials Physics, Anhui Key Lab of Nanomaterials and Nanotechnology, Institute of Solid State Physics, Hefei Institutes of Physical Science, Chinese Academy of Sciences, Hefei, Anhui, 230031, P. R. China.; 6 Tiangong University, Tianjin 300387, P. R. China.

**Keywords:** gold@manganese dioxide core-shell nanoparticles, radiotherapy, ROS amplification, hypoxia alleviation, MR-CT dual-modal imaging

## Abstract

**Rationale:** Radiotherapy is a principal modality in cancer treatment, effectively controlling local tumor growth and possessing the potential to enhance the immunogenicity of tumor cells, thereby improving the antitumor immunity. However, its efficacy is often limited by insufficient production of reactive oxygen species (ROS), tumor hypoxia, and the immunosuppressive tumor microenvironment (TME). Therefore, developing strategies to amplify ROS and reshaping the hypoxic, immunosuppressive TME is crucial for advancing radiotherapy.

**Methods:** In this study, we designed a polyethylene glycol (PEG)-modified gold@manganese dioxide core-shell nanoparticle (GMCN@PEG) that is responsive to the acidic TME. We then investigated its ability to enhance radiotherapy and magnetic resonance-computed tomography (MR-CT) dual-modality imaging both *in vivo* and *in vitro*.

**Results:** GMCN@PEG exhibits good biocompatibility under neutral physiological conditions and, upon exposure to the acidic TME, it alleviates tumor hypoxia and amplifies ROS production. This leads to enhanced radiotherapy sensitivity and the induction of immunogenic cell death (ICD). Furthermore, GMCN@PEG activates the cGAS-STING signaling pathway, promoting dendritic cells (DCs) maturation, macrophages M1 polarization, and T cells infiltration, effectively counteracting the immunosuppressive state within the TME. Additionally, GMCN@PEG enhances dual-modality imaging through MR-CT, achieving the integration of diagnosis and therapy.

**Conclusion:** In summary, GMCN@PEG as a multifunctional nanosensitizer, demonstrate significant potential and promise in improving the efficacy of radiotherapy, reshaping the tumor microenvironment, promoting antitumor immunity, and biomedical imaging enhancement.

## Introduction

Radiotherapy is a primary treatment modality for malignant solid tumors, which destroys cancer cells by directly inducing DNA breaks or by indirectly increasing the production of reactive oxygen species (ROS) within cancer cells 1. However, the rapid growth of cancer cells and twisted blood vessels can lead to hypoxia within solid tumors, and this hypoxia can enhance the ability of tumor cells to repair radiotherapy-induced DNA damage, leading to treatment resistance and radiotherapy failure 2,3. In addition, the degree of cancer cell damage during radiotherapy is highly dependent on ROS production 4. However, insufficient energy deposition, hypoxic microenvironment, and low radiation energy absorption coefficient lead to insufficient ROS production, which significantly reduces the efficacy of radiotherapy 5,6. To address these challenges, several clinical strategies have been explored. Hypoxia-targeting agents like nimorazole improve oxygen availability to enhance radiosensitivity 7,8, while DNA repair inhibitors (PARP inhibitors for homologous recombination deficiency, and DNA-dependent protein kinase inhibitors blocking non-homologous end joining) aim to sustain radiation-induced DNA damage 9,10**.** Despite progress, these strategies often focus on single molecular targets, failing to concurrently address TME heterogeneity and ROS-mediated radiosensitization. Therefore, improving the hypoxic tumor microenvironment and elevating ROS levels in tumor cells are crucial for enhancing the efficacy of radiotherapy.

Previous studies have demonstrated that radiotherapy not only effectively eliminates tumor cells directly and indirectly but also significantly modulates the immune response to tumors 11. While destroying tumor cells, high-energy radiation can trigger the release of tumor antigens, which in turn induces immunogenic cell death (ICD) and abscopal effects, stimulating a systemic immune response 12-14. Furthermore, radiotherapy can activate the cGAS-STING pathway by inducing double-stranded DNA breaks, upregulate the expression of type I interferons, and enhance the antitumor activity of T cells 15,16. However, radiotherapy alone is not sufficient to elicit a systemic antitumor immune response due to limited radiation absorption and the inherent immunosuppressive properties of the tumor microenvironment (TME) 17,18. Recently, nanoparticles have garnered significant interest in enhancing the efficacy of radiotherapy-induced immune response, yet most studies have only targeted antitumor immunity through a single pathway, underutilizing the potential of radiotherapy-induced immune response 19-22. Therefore, developing a novel nanoparticle capable of reshaping the immunosuppressed TME and enhancing radiotherapy-induced immune response through both ICD and cGAS-STING pathways is imperative.

**Scheme 1:**
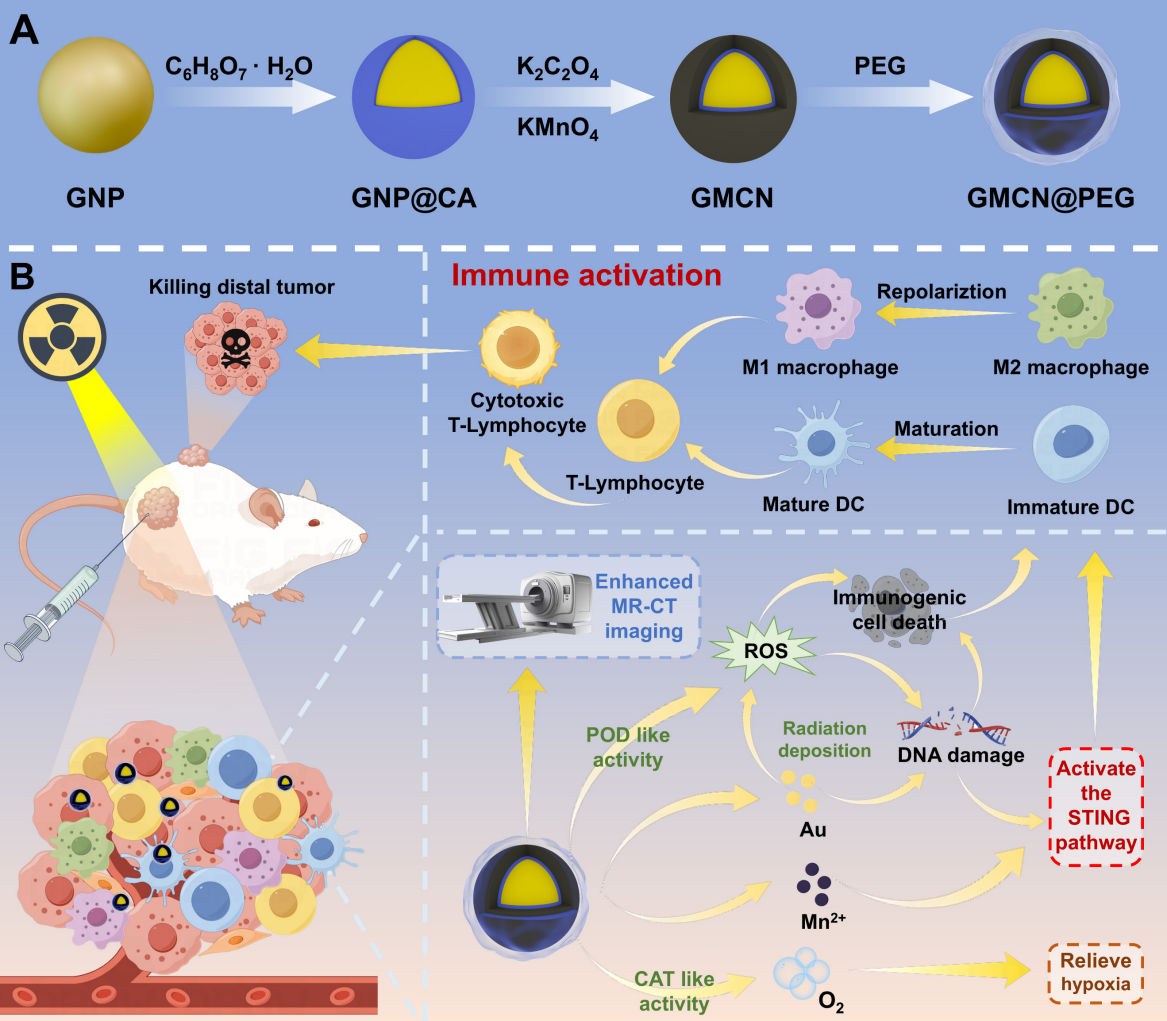
** Schematic illustration of the synthesis and therapeutic mechanism of GMCN@PEG for dual-modal MR-CT imaging and enhanced radiotherapy.** (A) Illustration of the preparation processes of GMCN@PEG. (B) Schematic demonstrated that GMCN@PEG combats primary and metastatic tumors by amplifying radiotherapy-induced ROS, boosting anti-tumor immunity, and alleviating hypoxia, while enabling MR-CT dual-modal imaging for integrated diagnosis and therapy.

In this study, we developed a core-shell nanostructure of gold nanoparticles coated with manganese dioxide (GMCN), which is proposed as nanosensitizer for enhanced radiotherapy-induced immune response and MR-CT dual-modal imaging (Scheme 1.). The GMCN surface, modified with polyethylene glycol (PEG), demonstrates high solubility and biocompatibility in neutral physiological conditions. These imaging and therapeutic capabilities are specifically activated under the acidic conditions of TME. The surface manganese dioxide (MnO_2_) catalyzes the decomposition of H_2_O_2_ into oxygen, alleviating hypoxia, and generates ROS and Mn^2+^ through peroxidase-like (POD-like) activity at acidic TME. The exposed gold nanoparticles (GNP) efficiently deposit radiation energy in the tumor, inducing ROS production and DNA double-strand breaks. The resulting ICD and the release of DNA fragments and Mn^2+^ activate the cGAS-STING signaling pathway, promoting DCs maturation and macrophages M1 polarization, thereby reshaping the immunosuppressed TME and enhancing the tumor immune response induced by radiotherapy. Additionally, the liberated Mn^2+^ and GNP enhance dual-modal MR-CT imaging, facilitating the integration of diagnosis and therapy.

## Results and Discussion

### Synthesis and Characterization of GMCN

As shown in Scheme 1, the formation process of gold@manganese dioxide core-shell nanoparticles (GMCN) should contain three steps. Initially, gold (Au) nanoparticles (GNP) are fabricated using a previously described method 23. In the subsequent step, the as-prepared GNP are subjected to a 12-hour incubation with citric acid (CA). This treatment modifies the surface of the GNP, facilitating the subsequent adsorption of manganese dioxide (MnO_2_). The final step involves the deposition of a MnO_2_ shell onto the surface of the CA-modified GNP. This is achieved by reducing potassium permanganate (KMnO_4_) in the presence of potassium oxalate (K_2_C_2_O_4_), resulting in the formation of GMCN.

**Figure 1:**
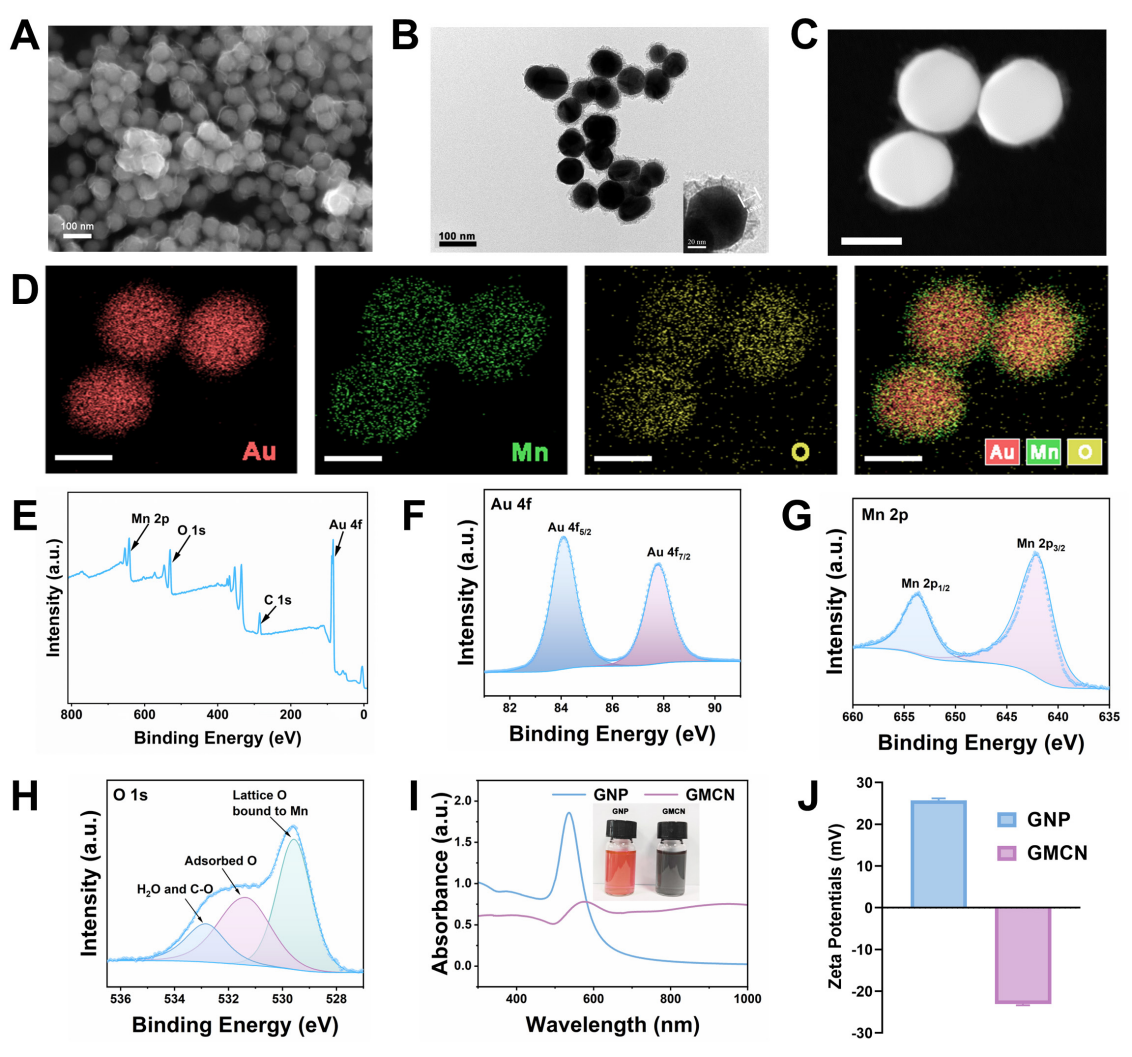
** Synthesis and characterization of GMCN.** (A) Representative SEM image of the GMCN. (B) Representative TEM image of the GMCN. (C) Representative HAADF-STEM image of GMCN. Scale bars:50 nm. (D) Elemental mapping of GMCN. Scale bars:50 nm. (E) Full-range survey XPS spectrum for GMCN. (F) Au 4f XPS spectrum for GMCN. (G) Mn 2p XPS spectrum for GMCN. (H) O 1s XPS spectrum for GMCN. (I) UV-vis-NIR absorption spectra of GNP and GMCN. (J) Zeta potential of GNP and GMCN.

Figure 1A and 1B present the scanning electron microscopy (SEM) and transmission electron microscopy (TEM) images, respectively, which clearly illustrate that the GMCN possesses a uniformly dispersed spherical morphology with a distinct core-shell structure. The core consists of gold nanoparticles, surrounded by a manganese dioxide shell with an approximate thickness of 15 nm. Energy dispersive X-ray spectroscopy (EDS) mapping of the GMCN further corroborates the core-shell architecture and elucidates the primary elemental composition, identifying gold (Au), manganese (Mn), and oxygen (O) as the predominant elements (Figure 1C-D, Figure S1A). Furthermore, the molar and mass ratios of the elements Au and Mn were analyzed by inductively coupled plasma-mass spectrometry (ICP-MS), with the results indicating a molar ratio of 2.96:1 and a mass ratio of 10.62:1, respectively (Figure S2A).

To study the chemical composition of GMCN, the X-ray photoelectron spectroscopy (XPS)-derived spectra for GMCN were obtained. The full-range survey of the XPS spectrum showed the presence of Au, Mn and O elements in GMCN, which is consistent with the results of the element mapping (Figure 1E). The high-resolution spectra of Au 4f, Mn 2p and O 1s are shown in Figure 1F-H. The two peaks of the Au 4f spectrum are located at 84.06 and 87.73 eV from Au 4f_5/2_ and Au 4f_7/2_, respectively (Figure 1F). Mn 2p also exhibits two distinct peaks located at 641.96ev and 653.72ev, which are attributed to the Mn 2p_1/2_ and Mn 2p_3/2_ spin-orbitals (Figure 1G). These peak positions are consistent with the characteristic peak positions of MnO_2_ 24,25, indicating that the Mn in GMCN is MnO_2_. Additionally, the O 1s spectra were examined, revealing three peaks (Figure 1H). The primary peak at 521.97 eV is assigned to the lattice oxygen bonding with Mn 26. A shoulder peak observed at 531.45 eV is likely due to oxygen species adsorbed onto the surface, and the weakest peak at the higher binding energy of 532.70 eV may be attributed to the O^2-^ of adsorbed H_2_O 27.

The UV-vis-NIR spectrum showed the characteristic absorption peaks of GMCN at 577 nm, which could be assigned to red-shifted peak of GNP in the formed GMCN (Figure 1I). Furthermore, zeta potential measurements uncovered a transformation in the surface charge of the GNP, transitioning from a negative to a positive polarity subsequent to the MnO_2_ coating process, thereby forming the GMCN (Figure 1J). Concurrently, Dynamic Light Scattering (DLS) studies yielded hydrated particle dimensions of 143.2 nm for the GNP and 205.5 nm for the GMCN, accompanied by polydispersity indices (PDI) of 0.010 and 0.161, respectively (Figure S2B). These values underscore the exceptional physical stability and dispersibility of the synthesized materials. The confluence of an enlarged particle size, the modification in UV-vis-NIR spectroscopic properties, and the observed decrease in zeta potential collectively constitutes robust evidence corroborating the successful synthesis of the GMCN.

### Enzyme-Like Activities of GMCN

**Figure 2:**
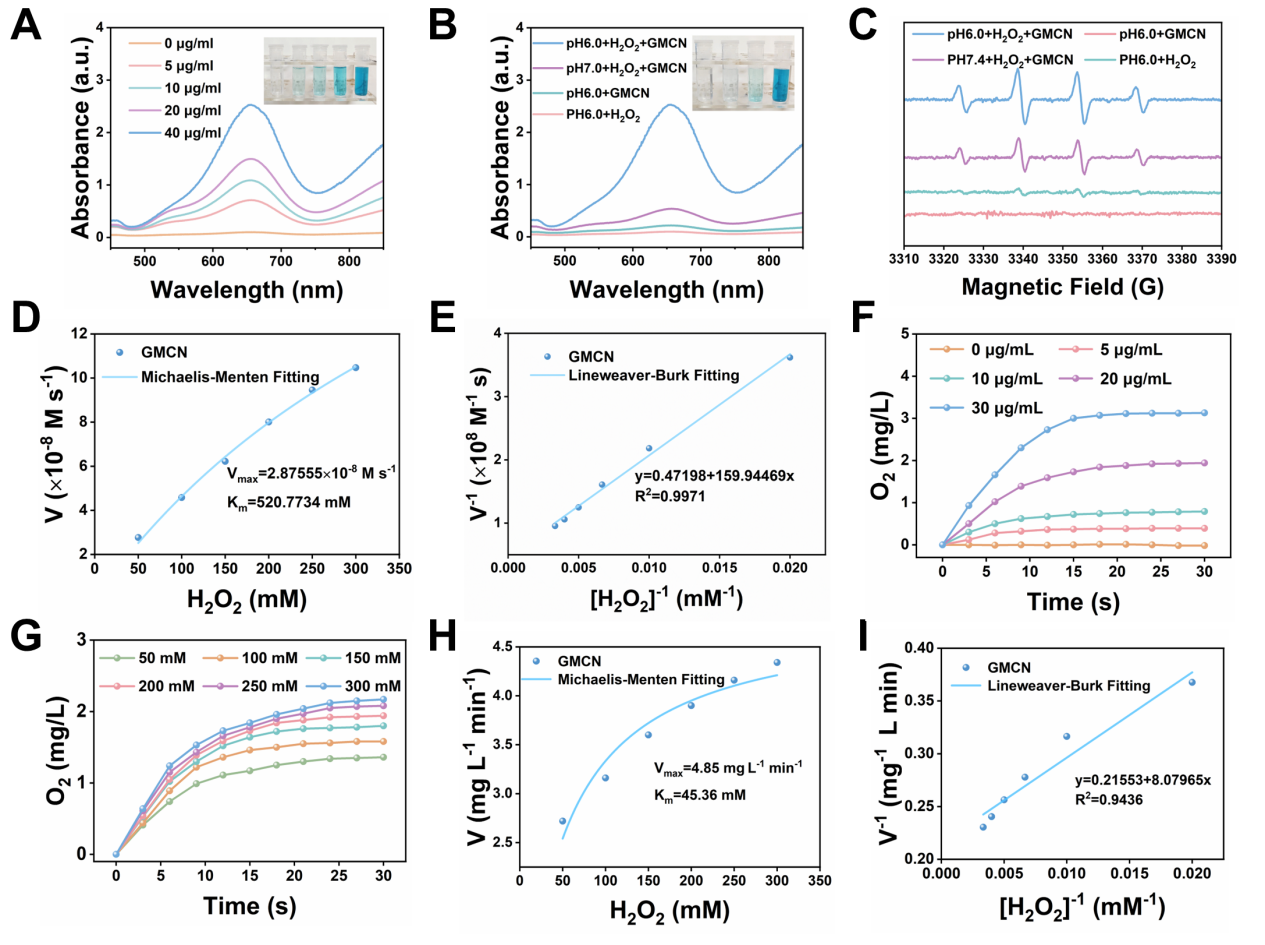
** Enzyme-like activities of GMCN.** (A) UV-vis absorption spectra of oxTMB upon the addition of H_2_O_2_ (100 mM) and different concentrations of GMCN at pH 6.0. (B) UV-vis absorption spectra of oxTMB upon with different conditions (GMCN = 40 μg mL^-1^, H_2_O_2_ = 100 mM). (C) ESR spectra for •OH at different conditions (GMCN = 40 μg mL^-1^, H_2_O_2_ = 100 mM). (D, E) POD-like activity-related Michaelis-Menten kinetic analysis (D) and Lineweaver-Burk plot (E) for GMCN with H_2_O_2_ as a substrate. (F) Production of O_2_ upon adding different concentrations of GMCN into H_2_O_2_ solution (200 mM) at pH 6.0. (G) Production of O_2_ upon adding GMCN (20 μg mL^-1^) into different concentrations of H_2_O_2_ solution at pH 6.0. (H, I) CAT-like activity-related Michaelis-Menten kinetic analysis (H) and Lineweaver-Burk plot (I) for GMCN with H_2_O_2_ as a substrate.

The hydroxyl radical (•OH) is one of the reactive oxygen species produced by ionizing radiation (IR) that leads to cell death 28. Many studies have reported that manganese-based oxides with peroxidase-like (POD-like) activity can catalyze the generation of •OH from H_2_O_2_ 29,30. In this study, we aimed to investigate the POD-like enzymatic activity of GMCN. The •OH derived from the catalytic reaction can transform 3,3′,5,5′-tetramethylbenzidine (TMB) into blue-colored oxidized TMB (oxTMB) with absorbance at 652 nm 31. Therefore, we evaluated the POD-like activity of GMCN using the colorimetric method of TMB. As show in Figure 2A, the intensity of the blue-colored solution deepened significantly with increasing GMCN concentration, correlating with the enhanced absorbance at 652 nm. Given that tumor tissues and cells are typically more acidic than normal tissues, we analyzed the POD-like activity of GMCN under different pH conditions 32. Interestingly, GMCN exhibited moderate POD-like activity in weakly alkaline conditions (pH 7.4), while its enzymatic activity significantly increased in weakly acidic conditions (pH 6.0) (Figure 2B). Additionally, we utilized the electron spin resonance (ESR) technique to detect radical adducts between the generated •OH and the spin-trap molecule 5,5-dimethyl-1-pyrroline N-oxide (DMPO). As show in Figure 2C, the ESR signal intensities of •OH (a quartet signal of 1:2:2:1) were stronger in weakly acidic conditions (pH 6.0) than in weakly alkaline conditions (pH 7.4), indicating that the POD-like activity of GMCN is pH-dependent. Furthermore, we conducted steady-state catalytic kinetic analyses to systematically evaluate the POD-like catalytic performance of GMCN (Figure 2D-E). Subsequently, we calculated the initial reaction rates (v_0_) according to the Beer-Lambert law and determined the V_max_ (the maximum reaction rate when the enzyme is saturated with the substrate) and K_m_ (the affinity of an enzyme for its substrate) values as previously mentioned. The V_max_ and K_m_ for the POD-like activity were found to be 2.88 × 10^-8^ M s^-1^ and 520.77 mM, respectively (Figure 2D).

Hypoxia is a prominent feature of the tumor microenvironment and a key factor contributing to tumor resistance to radiotherapy 33. Many studies have suggested that alleviating the hypoxic status of the tumor microenvironment can enhance the sensitivity of radiotherapy 34,35. To investigate the catalase-like (CAT-like) activity of GMCN, which can decompose H_2_O_2_ into H_2_O and O_2_, we employed a dissolved oxygen meter to monitor the production of O_2_. As shown in Figure 2F, the catalytic efficiency of GMCN displayed a clear concentration dependence. To further evaluate the CAT-like activity of GMCN, we performed a steady-state kinetic analysis. After adding GMCN (20 μg mL^-1^) and varying concentrations of H_2_O_2_ in an acidic PBS solution (pH 6.0), we recorded the time-dependence, H_2_O_2_ concentration-dependence, and the amount of O_2_ generated. The initial reaction rates (v_0_) under different H_2_O_2_ concentrations were evaluated (Figure 2G). Subsequently, the reaction rates were plotted relative to the corresponding H_2_O_2_ concentrations and fitted using the Michaelis-Menten saturation curve (Figure 2H-I). Upon calculation, the V_max_ and K_m_ for GMCN were determined as 4.85 mg L^-1^ min^-1^ and 45.36 mM, respectively (Figure 2H).

### Radiotherapy Sensitization of GMCN@PEG *In Vitro*

To augment the biocompatibility of the GMCN and attenuate their potential toxicity in both *in vitro* cellular assays and *in vivo* animal models, a polyethylene glycol (PEG) layer was conjugated onto the nanoparticle surfaces. As depicted in Figure S3A and S3B, the observed changes in particle dimensions and zeta potential serve as indicative markers of the successful PEGylation process. The cytotoxicity assays, conducted using the Cell Counting Kit-8 (CCK8), revealed a significant reduction in the cytotoxic effects of the PEGylated GMCN and GNP, as compared to their non-modified counterparts (Figure S3C-D). Consequently, both GNP and GMCN underwent PEG modification in subsequent cell and animal experiments.

Furthermore, biotransmission electron microscopy (Bio-TEM) revealed effective internalization of GMCN@PEG by 4T1 cells (Figure S4A), a critical step for its tumor-killing function. Significantly, under weakly acidic conditions (pH 6.0), TEM demonstrated disintegration of the MnO₂ shell in GMCN@PEG (Figure S4B), confirming its responsiveness to the acidic tumor microenvironment. This pH-dependent degradation could facilitate targeted tumor cell killing.

**Figure 3:**
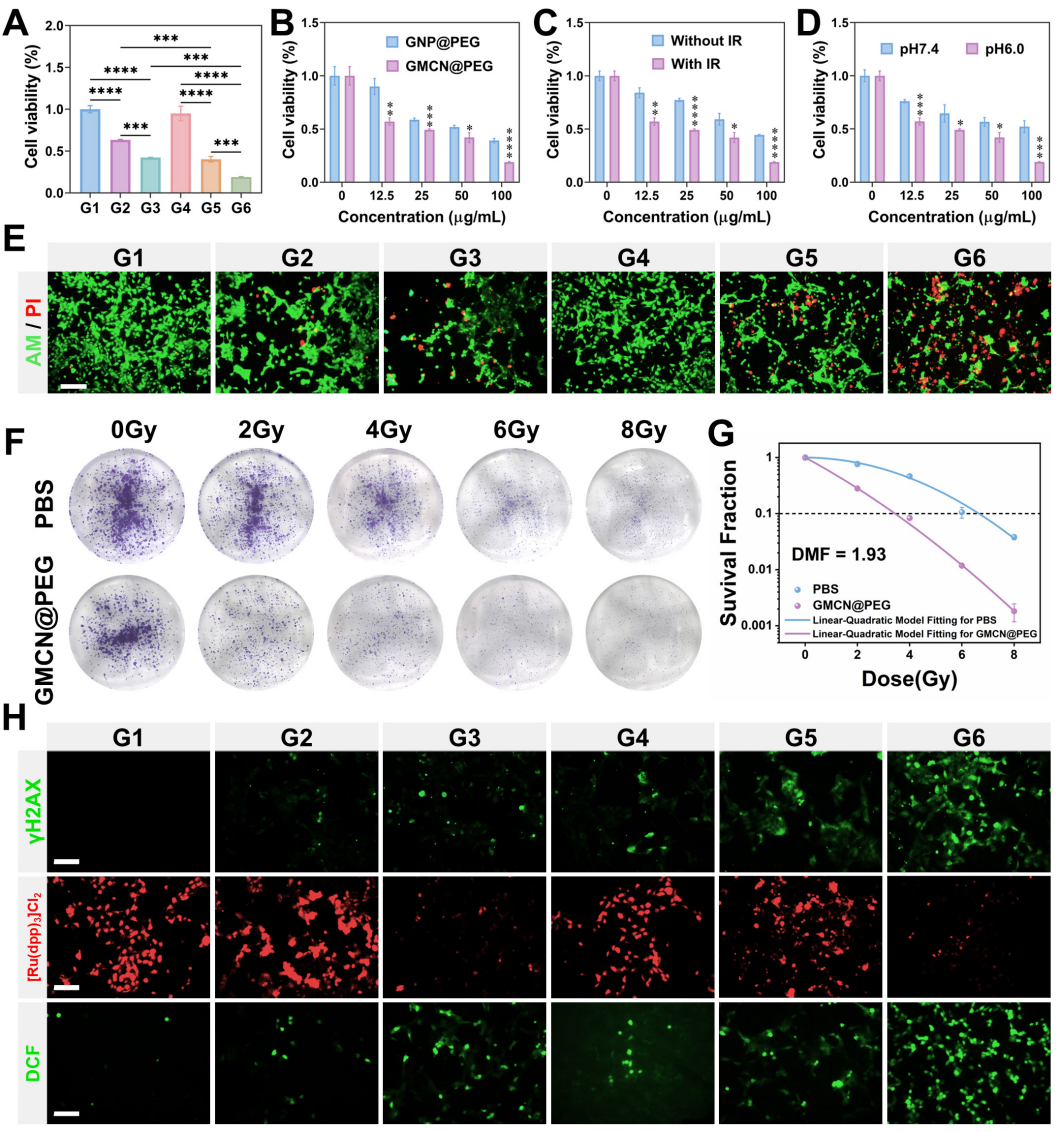
** Radiotherapy sensitization of GMCN@PEG *in vitro*** (A) Viability of 4T1 cells with different treated groups at pH 6.0. G1: Control; G2: GNP@PEG; G3: GMCN@PEG; G4: IR; G5: IR+GNP@PEG; G6: IR+GMCN@PEG. (B) Viability of 4T1 cells treated with different concentrations of GNP@PEG and GMCN@PEG under IR exposure. (C) Viability of 4T1 cells treated with different concentrations of GMCN@PEG, with or without IR exposure (D) Viability of 4T1 cells treated with different concentrations of GMCN@PEG at pH 6.0 versus pH 7.4. (E) Confocal fluorescence microscopy images of 4T1 cells stained with calcein-AM/PI, representing different treatment groups at pH 6.0. Scale bar: 100 µm. (F) Representative colonies of 4T1 cells treated with PBS and GMCN@PEG under various X-ray doses. (G) Clonogenic survival curves of 4T1 cells treat with PBS and GMCN@PEG under various X-ray doses. (H) Confocal fluorescence microscopy images of 4T1 cells stained for γH2AX, [Ru(dpp)_3_Cl_2_], and DCFH-DA, representing different treatment groups at pH 6.0. Scale bar: 100 µm. Statistical significance is indicated by asterisks: *p < 0.05, **p < 0.01, ***p < 0.001, ****p < 0.0001. Data are presented as means ± SD.

In an effort to elucidate the radiosensitizing potential of GMCN@PEG, we conducted a series of experiments with 4T1 cells under varying pH conditions and GMCN@PEG concentrations. The cells were exposed to GMCN@PEG at concentrations ranging from 0 to 100 μg mL^-1^ for 12 hours under both weakly alkaline (pH 7.4) and acidic (pH 6.0) conditions, followed by irradiation with an 8 Gy dose of X-rays. Subsequently, cell viability was assessed using the CCK8 assay after an additional 12-hour incubation period. Our findings indicate that both GMCN@PEG and GNP@PEG significantly potentiated the cytotoxic effects of radiotherapy, particularly under acidic conditions, where an inverse relationship between cell survival and GMCN@PEG concentration was observed (Figure 3A-B, Figure S5A-B). Notably, GMCN@PEG demonstrated a superior radiosensitizing effect compared to GNP@PEG, as evidenced by a markedly reduced cell survival rate (Figure 3C, Figure S5C). Moreover, the radiosensitizing efficacy of GMCN@PEG was found to be pH-dependent, with diminished effects under weakly alkaline conditions (Figure 3D). To further corroborate these findings, we employed Calcein-AM/PI staining to visualize cell survival, revealing that GMCN@PEG significantly increased the number of apoptotic cells (indicated by red fluorescence) post-irradiation, thereby augmenting the therapeutic impact of radiotherapy (Figure 3E, Figure S5G). Further validation was sought through clone formation assays, which revealed that GMCN@PEG and GNP@PEG both exhibited radiosensitizing properties (Figure 3F, Figure S5D). Specifically, the dose modification factor (DMF) at a 10% survival fraction for GMCN@PEG was calculated to be 1.93, surpassing that of GNP@PEG at 1.12, underscoring the enhanced radiosensitizing capability of GMCN@PEG (Figure 3G, Figure S5E).

The biological underpinnings of radiotherapy-induced tumor cell death involve the induction of DNA double-strand breaks and the promotion of reactive oxygen species (ROS) production within cancer cells 1. To explore the impact of GMCN@PEG on these parameters, we utilized fluorescence imaging to assess DNA damage and ROS levels post-irradiation. The results demonstrated a significant increase in both DNA double-strand breaks and ROS levels in the group treated with IR+GMCN@PEG, as compared to the control, GNP@PEG, GMCN@PEG, IR, and IR+GNP@PEG groups (Figure 3H, Figure S5F-G, Figure S6A). Furthermore, recognizing that radiotherapy can exacerbate tumor hypoxia, thereby reducing oxygen-dependent DNA damage and inducing hypoxia-inducible factor-1 alpha (HIF-1A)-mediated cell survival, we investigated the capacity of GMCN@PEG to mitigate tumor hypoxia 36. Utilizing [Ru(dpp)_3_]Cl_2_, an oxygen-sensitive fluorescent probe, we observed that the addition of GMCN@PEG led to a significant reduction in the probe's red fluorescence intensity, indicative of enhanced oxygen availability within the tumor microenvironment (Figure 3H) 37,38.

### GMCN@PEG Enhances Radiotherapy-Induced Immune Response *In Vitro*

In recent years, extensive research has underscored the dual role of radiotherapy in combating tumors-directly targeting tumor cells and modulating the immune response against them 39,40. High-energy radiation triggers the release of tumor antigens upon tumor cell demise, thereby initiating ICD 41. This process hinges on the liberation of damage-associated molecular patterns (DAMPs) from tumor cells, such as surface-exposed calreticulin (CRT) and high mobility group box 1 protein (HMGB1), which play pivotal roles in recruiting and maturing antigen-presenting cells (APCs) 42. Concurrently, reactive oxygen species (ROS) and other oxidative stressors are known to induce endoplasmic reticulum stress, further facilitating tumor ICD 43.

**Figure 4:**
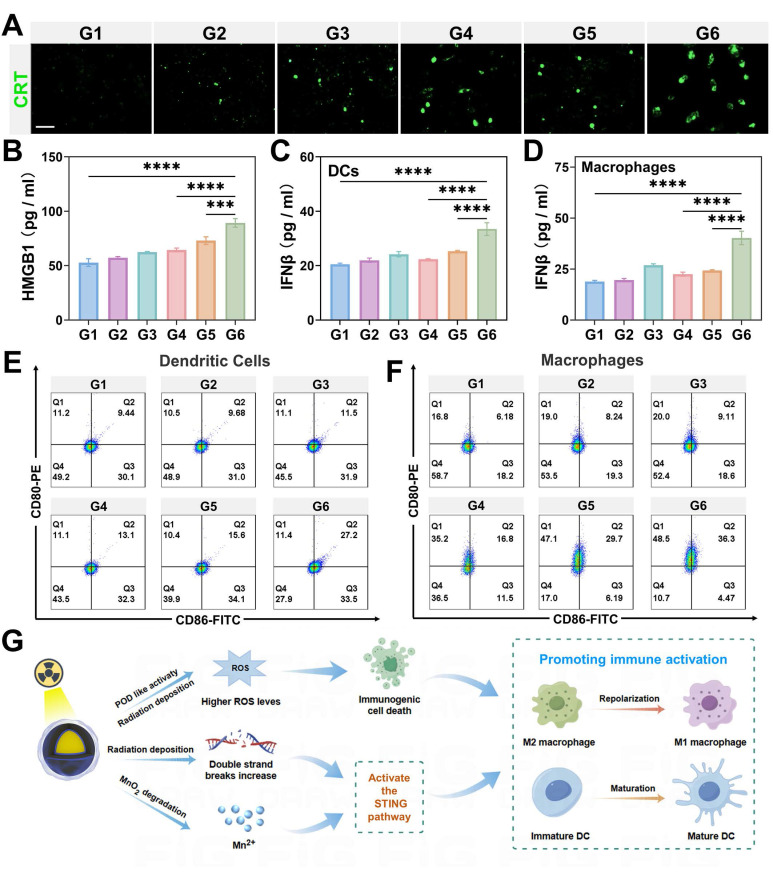
** GMCN@PEG enhances radiotherapy-induced immune response *in vitro*.** (A) Confocal fluorescence microscopy images of 4T1 cells immunofluorescence-stained for CRT, representing different treatment groups under acidic conditions (pH 6.0). G1: Control; G2: GNP@PEG; G3: GMCN@PEG; G4: IR; G5: IR+GNP@PEG; G6: IR+GMCN@PEG. Scale bar: 100 µm. (B-D) Quantitative analysis of the cytokines secreted by 4T1 cells (B), DCs (C) and macrophages (D) in the medium after treatment with different groups under acidic conditions. (E, F) Flow cytometry analysis of the co-expression of CD80 and CD86 in DCs (E) and macrophages (F) treated with different groups under acidic conditions. (G) Schematic illustration depicting the role of GMCN@PEG in enhancing radiotherapy-induced immune response. Statistical significance is indicated by asterisks: *p < 0.05, **p < 0.01, ***p < 0.001, ****p < 0.0001. Data are presented as means ± SD.

In our study, we observed that GMCN@PEG significantly amplifies ROS production. Accordingly, we delved deeper into the synergistic impact of GMCN@PEG on radiotherapy-induced ICD. Utilizing immunofluorescence staining and flow cytometry, we assessed CRT expression on the surface of 4T1 cells-a recognized marker of ICD. Our results distinctly revealed heightened CRT expression in the IR+GMCN@PEG group compared to control, GNP@PEG, GMCN@PEG, IR alone, and IR+GMCN@PEG groups (Figure 4A, Figure S7A-B). Furthermore, ELISA analysis of HMGB1-a crucial ICD marker-indicated its highest concentration in the supernatant of 4T1 cells treated with IR+GMCN@PEG (Figure 4B). These findings collectively highlight the potent enhancement of radiotherapy-induced immunogenic cell death by GMCN@PEG.

Another key mechanism by which radiotherapy promotes antitumor immune response is through activation of the cGAS-STING signaling pathway, triggering type I interferon cascade 44. Type I interferon signaling plays a crucial role in activating DCs, which, upon maturation, efficiently present antigens to T lymphocytes 45. Additionally, recent studies suggest that Mn^2+^ acts as an agonist for the cGAS-STING pathway, stimulating DCs maturation and polarization of macrophages towards the M1 phenotype, thereby enhancing antitumor immunity 46,47. Here, we assessed the secretion of type I interferon IFN-β in DCs and macrophages from different treatment groups to evaluate the ability of GMCN@PEG to promote activation of the STING signaling pathway during radiotherapy. The results, as shown in the Figure 4C and 4D, indicate a significant increase in IFN-β expression in cells treated with IR+GMCN@PEG compared to other treatment groups, suggesting that the combination of IR and GMCN@PEG can significantly enhance activation of the cGAS-STING signaling pathway. Furthermore, Western blot analysis revealed enhanced activation of the cGAS-STING pathway. Compared to radiotherapy alone, the GMCN@PEG combination group exhibited significantly increased phosphorylation of STING (p-STING), IRF3 (p-IRF3), and TBK1 (p-TBK1), demonstrating GMCN@PEG's ability to potentiate radiotherapy-induced cGAS-STING signaling (Figure S7D-G).

ICD and activation of the STING signaling pathway both promote DCs maturation and polarization of macrophages towards the M1 phenotype 48,49. Therefore, we investigated the effects of IR and GMCN@PEG co-treatment on the maturation of DCs and the M1 polarization profile of macrophages. Utilizing flow cytometry, we assessed the expression of CD80 and CD86, which are quintessential markers indicative of mature DCs and M1 macrophages, respectively 27. Our findings, as delineated in Figure 4E-F and Figure S7C, reveal a pronounced augmentation in the frequency of mature DCs characterized by the co-expression of CD80 and CD86, as well as an elevated proportion of M1 macrophages marked by the same phenotypic markers, within the group subjected to the combined regimen of irradiation (IR) and application of GMCN@PEG, when juxtaposed with other treatment groups. This observation underscores the synergistic potential of IR and GMCN@PEG in modulating the immune microenvironment, thereby augmenting the immunogenicity of dying tumor cells and bolstering the host's immune response.

Significantly, we observed that GNP@PEG enhances radiation-induced ICD and activates the cGAS-STING signaling pathway. This effect is likely due to the efficient deposition of radiation energy into tumor regions by GNP, leading to increased intracellular ROS levels and DNA double-strand breaks, thereby promoting radiation-induced ICD and cGAS-STING pathway activation 50. In contrast, GMCN@PEG demonstrates more pronounced effects by enhancing radiation deposition within tumor sites, further elevating ROS levels through its POD-like and CAT-like activities, ameliorating hypoxia, and intensifying DNA double-strand breaks (Figure 4G). Moreover, Mn^2+^ functions as a STING agonist, thereby augmenting antitumor immune responses (Figure 4G).

### Biological Safety and Biocompatibility of GMCN@PEG* In Vivo*

**Figure 5:**
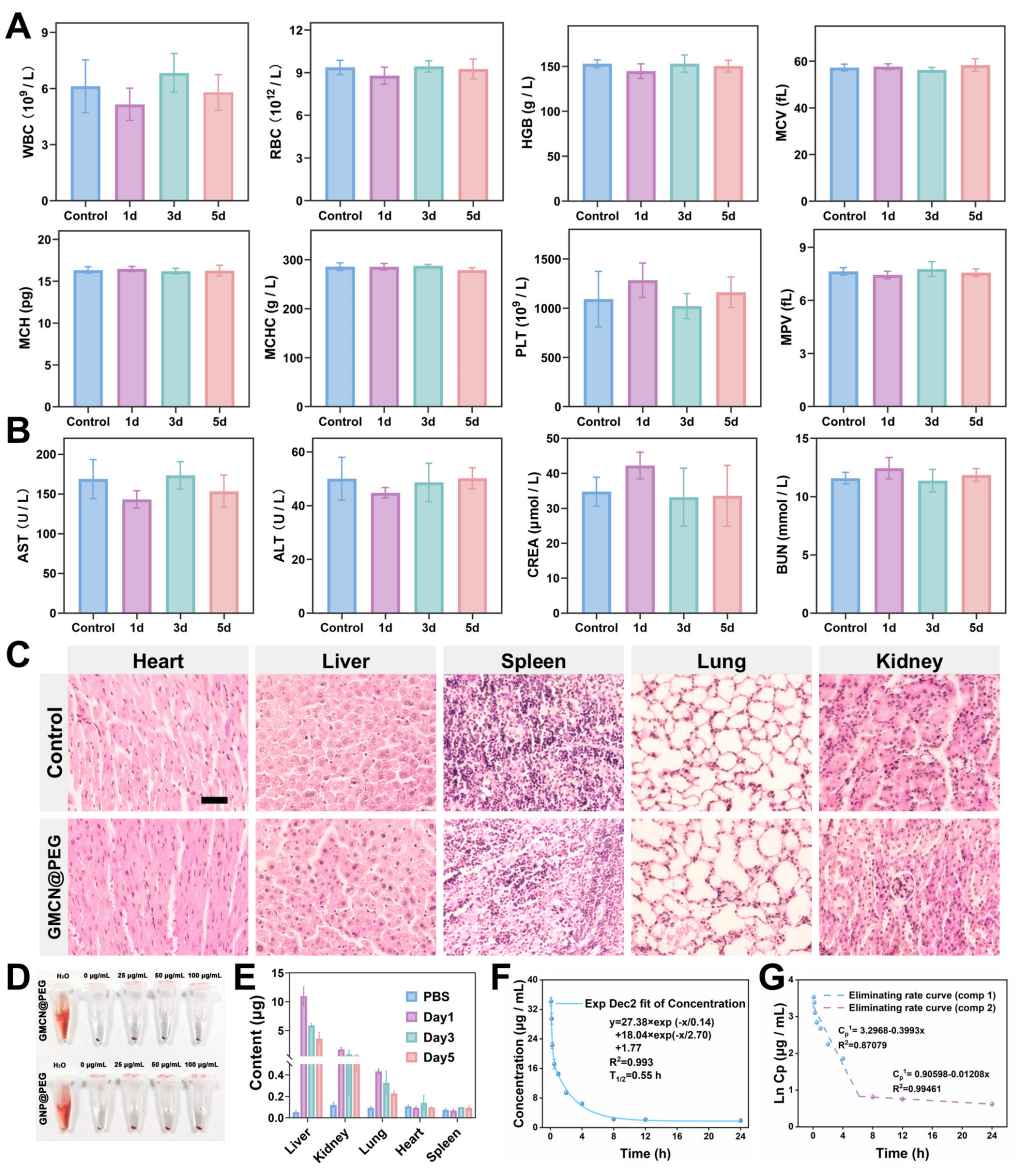
** Biological safety and biocompatibility of GMCN@PEG *in vivo*.** (A, B) Blood routine and biochemical analyses in mice following intravenous injection of PBS or GMCN@PEG at days 1, 3, and 5 post-injection. (WBC, white blood cell; RBC, red blood cell; HGB, hemoglobin; MCV, mean corpuscular volume; MCH, mean corpuscular hemoglobin; MCHC, mean corpuscular hemoglobin concentration; PLT, platelet; MPV, mean platelet volume; ALT, alanine aminotransferase; AST, aspartate aminotransferase; BUN, blood urea nitrogen; CREA, Creatinine). (C) H&E staining of tissue sections from major organs of mice injected with PBS or GMCN@PEG for 5 days. Scale bar: 100 µm. (D) Evaluation of the hemolytic properties of GNP@PEG and GMCN@PEG. (E) Biodistribution of GMCN@PEG in major organs of mice following intravenous injection at various time points. (F) Blood circulation curve of mice intravenously injected with GMCN@PEG. (G) The eliminating rate curve of intravenously injected GMCN@PEG from the blood circulation curve according to the ln(concentration)-T relationship.

Based on the findings obtained at the cellular level, the biological safety, pharmacokinetics, and biodistribution of GMCN@PEG were explored *in vivo*. For evaluating the biological safety, 5-week-old healthy female ICR mice were intravenously injected with GMCN@PEG via the tail vein on days 1, 3, and 5. Subsequently, blood samples were collected from the mice for hematological and biochemical analyses. Analysis revealed no statistically significant variances in hematologic parameters, liver function, or renal function indices between mice injected with GMCN@PEG on the specified days and the control group (Figure 5A-B). Furthermore, histological examination of tissue sections from major organs showed no signs of inflammation, hemorrhage, necrosis, or other abnormalities (Figure 5C). Additionally, incubation of nanoparticles with mouse-derived erythrocytes for 12 hours did not induce significant hemolysis, underscoring its excellent blood compatibility (Figure 5D). We also assessed the long-term stability of GMCN@PEG in physiological environments. The particle size and polydispersity index (PDI) remained largely unchanged in phosphate-buffered saline (PBS), RPMI-1640 medium supplemented with 10% fetal bovine serum (RPMI-1640+10%FBS), and fetal bovine serum (FBS), as demonstrated in the Figure S8A-F. These observations suggest that GMCN@PEG maintains its stability in physiological conditions over an extended period. In summary, our findings suggest that GMCN@PEG demonstrates favorable biosafety *in vivo*.

Following the evaluation of biological safety, we conducted an analysis of the pharmacokinetic profile of GMCN@PEG to investigate it *in vivo* metabolic behavior. GMCN@PEG was administered via the tail vein on days 1, 3, and 5, and the gold content in various organs was quantified using ICP-OES to determine the biodistribution of GMCN@PEG across major organs and tumor at different time points. The results are depicted in Figure 5E and Figure S8G, consistent with the behavior of many nanomaterials, GMCN@PEG exhibited preferential accumulation in the liver and kidneys, potentially attributed to reticuloendothelial system entrapment. In tumors, accumulation peaks approximately 1 h post-injection but subsequently declines due to metabolic clearance (Figure S8G). This rapid clearance represents a main challenge in maintaining sustained therapeutic concentrations through nanomedicine. Furthermore, a two-compartment model was employed to simulate the circulation of GMCN@PEG within the bloodstream, yielding a calculated circulating half-life of 0.55 hours (Figure 5F). By analyzing the Ln(concentration)-time relationship, we determined the elimination rate constant of GMCN@PEG to be -0.3993 μg mL^-1^ h^-1^ in the initial phase and 0.01208 μg mL^-1^ h^-1^ in the subsequent phase (Figure 5G) 51.

### MR-CT Dual-Mode Imaging Enhancement of GMCN@PEG

**Figure 6:**
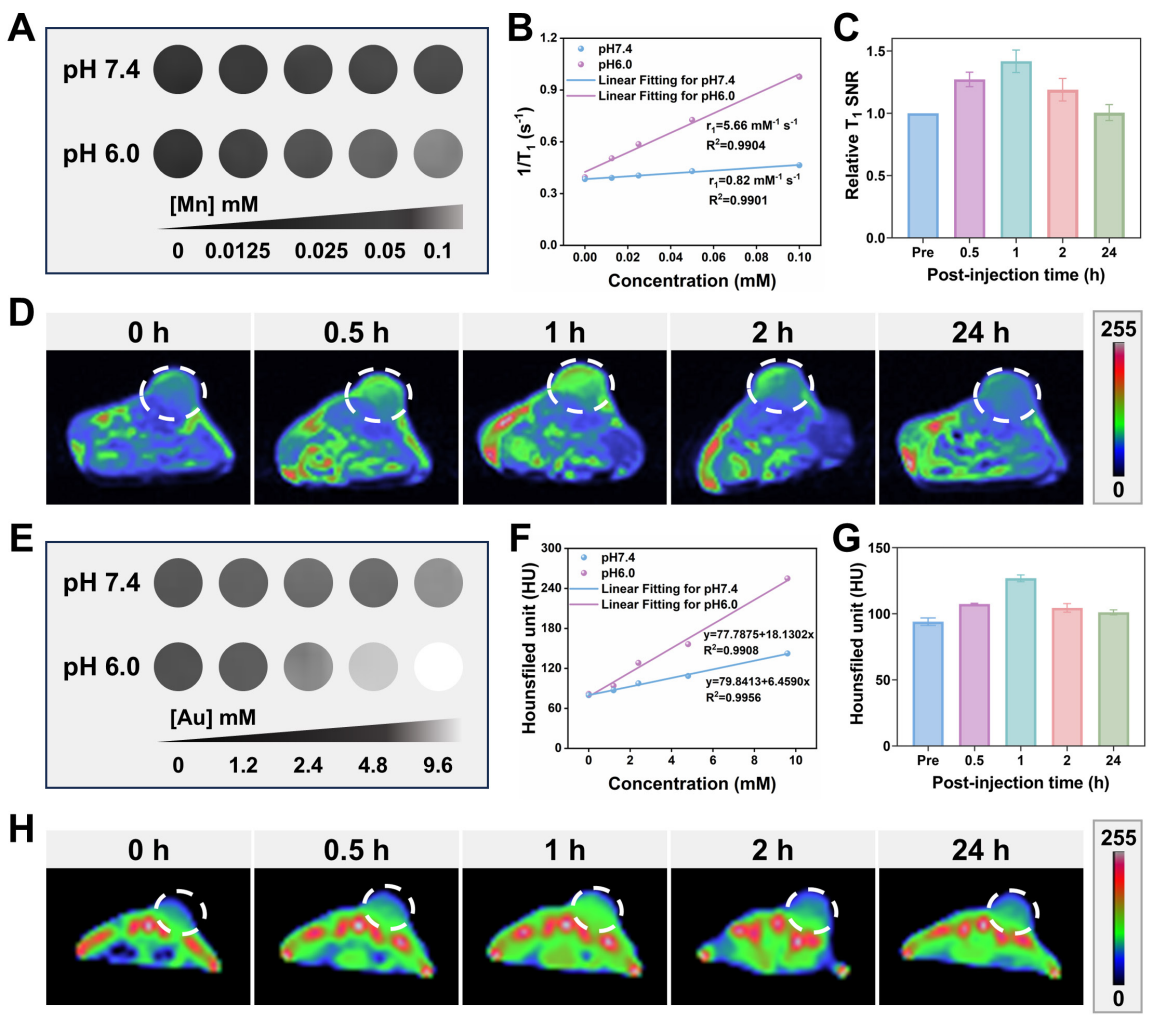
** MR-CT dual-mode imaging enhancement of GMCN@PEG.** (A) T_1_-weighted MRI of a GMCN@PEG aqueous solution with different [Mn] concentrations at pH 6.0 or 7.4. (B) The linear relationship between T_1_ longitudinal relaxation rate and [Mn] concentration at pH 6.0 or 7.4. (C) Relative T_1_ signal-to-noise ratio (SNR) of 4T1 tumor bearing BALB/c mice after i.v. injection of GMCN@PEG (10 mg kg^-1^) at various time points. (D) T_1_-weighted MR pseudo-color images of 4T1 tumor bearing BALB/c mice after i.v. injection of GMCN@PEG (10 mg kg^-1^) at various time points. (E) CT images of a GMCN@PEG aqueous solution with different [Mn] concentrations at pH 6.0 or 7.4. (F) The linear relationship between CT value and [Mn] concentration at pH 6.0 or 7.4. (G) CT value of 4T1 tumor bearing BALB/c mice after i.v. injection of GMCN@PEG (10 mg kg^-1^) at various time points. (H) CT pseudo-color images of 4T1 tumor bearing BALB/c mice after i.v. injection of GMCN@PEG (10 mg kg^-1^) at various time points.

Considering the capability of the GMCN@PEG would be decomposed to generate Mn^2+^ based on the acidic TME, the imaging property of the GMCN@PEG was explored 52. As shown in Figure 6A, we observed a [Mn] concentration-dependent effect on the T_1_ signal under acidic conditions (pH 6.0), and there was no noticeable increase in the T_1_ signal under neutral conditions (pH 7.4). However, with the decline in PH, the T_1_ relaxivity dramatically increased from 0.82 to 5.66 mM^-1^ s^-1^ (Figure 6B). Supported by the acidic TME in tumor tissues, the GMCN@PEG also showed good performance in MRI *in vivo*. As shown in Figure 6C-D and Figure S5B, the T_1_-weighted imaging signal in the tumor region was significantly enhanced after the injection of GMCN@PEG compared with that before the intravenous injection of GMCN@PEG, which was maximal at 60 min. the T_1_ signals began to decay after 90 min because GMCN@PEG began to be metabolized by the mice. These results suggest that GMCN@PEG has an acidic-responsive T_1_-MRI contrast ability.

Upon the disintegration of the MnO_2_ shells in GMCN@PEG, the gold cores are exposed, enhancing radiosensitivity and exhibiting superior X-ray attenuation. This renders them suitable for CT imaging 53. Figure 6E and 6F present the corresponding CT images of the GMCN@PEG aqueous solution at varying pH levels. At pH 6.0, the grayscale levels transition from dark to light as the sample concentration increases, suggesting that higher sample concentrations are associated with stronger X-ray absorption and improved contrast agent imaging efficacy. *In vivo* CT imaging of GMCN@PEG with mice demonstrated good X-ray attenuation. As depicted in Figures 6G-H and Figure S5B, CT imaging signals were significantly enhanced post-GMCN@PEG injection in tumor-bearing mice, peaking at 1 hour, which coincided with the T_1_-weighted MR imaging signal. Collectively, these findings underscore the acidic TME-responsive dual-modality MR-CT imaging capabilities of GMCN@PEG.

Furthermore, we evaluated GMCN@PEG against clinically available contrast agents. As demonstrated in Figure S9C-E, GMCN@PEG achieves a higher relative T1-weighted signal-to-noise ratio (SNR) than gadopentate dimeglumine, indicating superior T1 contrast enhancement. For CT imaging, GMCN@PEG generates higher Hounsfield unit (HU) values, yielding enhanced contrast resolution (Figure S9F-H). Collectively, GMCN@PEG outperforms commercial contrast agents in both MR and CT imaging modalities while enabling integrated MR-CT dual-modal imaging.

### GMCN@PEG for Radiotherapy Enhancement in Primary Tumor-Bearing Mice

**Figure 7:**
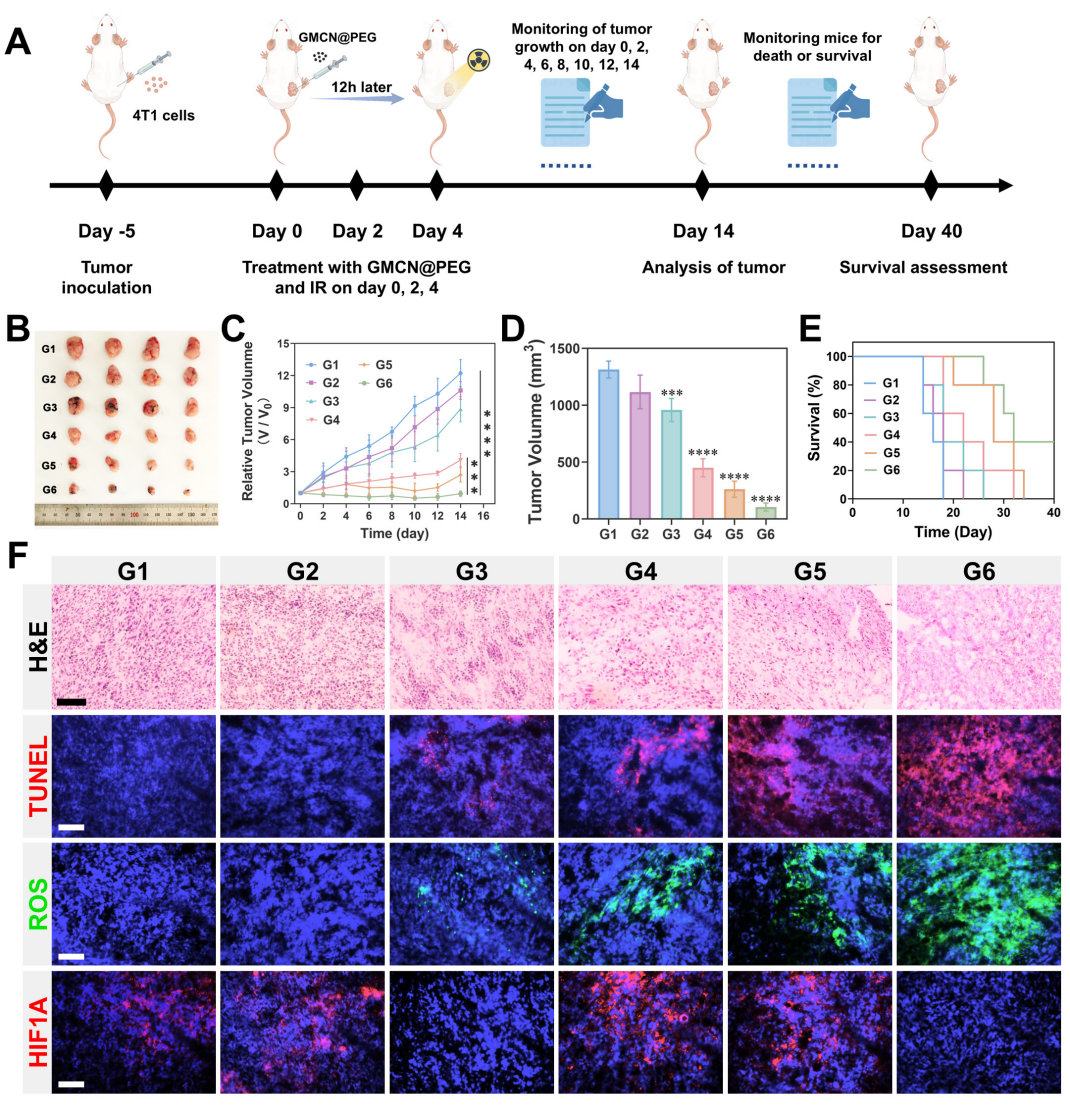
** GMCN@PEG for radiotherapy enhancement in primary tumor-bearing mice** (A) Illustration of primary 4T1 tumor therapy *in vivo*. (B) The image of the dissected primary tumors from each group on day 14. G1: Control; G2: GNP@PEG; G3: GMCN@PEG; G4: IR; G5: IR+GNP@PEG; G6: IR+GMCN@PEG. (C) Average tumor growth curves of primary tumors after various treatments. (D) Average tumor volume of primary tumors from each group on day 14. (E) Kaplan-Meier survival curves of the mice after the indicated treatments. (F) Histological analysis with H&E, TUNEL, ROS, and HIF1A staining of primary tumor sections from each treatment group on day 14. Scale bar: 100 µm. Statistical significance is indicated by asterisks: *p < 0.05, **p < 0.01, ***p < 0.001, ****p < 0.0001. Data are presented as means ± SD.

Given the excellent therapeutic efficacy *in vitro* and favorable biocompatibility of GMCN@PEG, we conducted an assessment of its *in vivo* antitumor effects utilizing a 4T1 subcutaneous tumor model in murine subjects. The tumor-bearing mice were randomly divided into six groups: Control, GNP@PEG, GMCN@PEG, IR, IR+GNP@PEG, and IR+GMCN@PEG. Saline, GNP@PEG, and GMCN@PEG were administered via intratumoral injection at a dose of 10 mg kg^-1^. Subsequent to the GMCN@PEG injection, radiotherapy was administered 12 hours later employing a medical X-ray irradiator at an 8 Gy dose, with a source-to-surface distance set at 100 cm. The treatment cycle is shown in Figure 7A. The tumors' growth and mice's body weight were monitored (Figure 7B-D, Figure S10A). As expected, mice in the control group had the fastest growing tumors, which grew about 12-fold and reached a volume of 1300 mm³, while each treatment group displayed varying degrees of tumor growth suppression. Notably, the IR+GMCN@PEG group demonstrated the most substantial reduction, with a tumor volume of merely 104 mm³. Furthermore, the IR+GMCN@PEG cohort exhibited markedly enhanced survival rates, with 40% of the murine subjects surviving at the 40-day mark, surpassing the survival durations of the other treatment groups (Control: 18 days, GNP@PEG: 22 days, GMCN@PEG: 26 days, IR: 32 days, IR+GNP@PEG: 34 days) (Figure 7E). These findings underscore the capacity of GMCN@PEG as a radiosensitizer to effectively impede tumor growth.

The tumor-killing efficacy of each treatment group was further assessed through histopathological examinations (Figure 7F). Hematoxylin and eosin (H&E) staining unveiled nuclear condensation or absence in tumor sections from the IR+GMCN@PEG group, while TdT-mediated dUTP nick-end labeling (TUNEL) staining indicated a significant increase in apoptosis of tumor cells following radiotherapy in the presence of GMCN@PEG, thus corroborating the enhanced tumor-killing effects of GMCN@PEG. Hypoxia-inducible factor-1 alpha (HIF-1A), activated under tumor hypoxic conditions, serves as a marker for assessing tumor hypoxia 54. Immunofluorescence analysis of HIF-1A expression exhibited a marked decrease in tumors of mice treated with GMCN@PEG, suggesting the CAT-like activity of GMCN@PEG in ameliorating tumor hypoxia. GMCN@PEG-mediated alleviation of tumor hypoxia, as detected by Hypoxyprobe™-1 Plus Kit, further validates the nanomaterial's CAT-like enzymatic activity (Figure S10B). Evaluation of ROS levels in tumor cells using DCFH-DA fluorescence probes demonstrated a significant elevation in ROS levels in the IR+GMCN@PEG group, confirming the POD-like activity of GMCN@PEG. These findings provide strong support for GMCN@PEG to enhance the efficacy of tumor radiotherapy.

Furthermore, we evaluated the therapeutic efficacy of intravenously administered GMCN@PEG. As shown in Figure S11A-D, GMCN@PEG significantly inhibited tumor growth. Notably, when combined with radiotherapy, GMCN@PEG synergistically enhanced tumor suppression. Despite its transient tumor accumulation profile, GMCN@PEG demonstrated potent radiosensitizing effects. These findings highlight the potential utility of GMCN@PEG as a radiotherapy enhancer.

### GMCN@PEG Enhances Radiotherapy-Induced Immune Response *in vivo*

**Figure 8:**
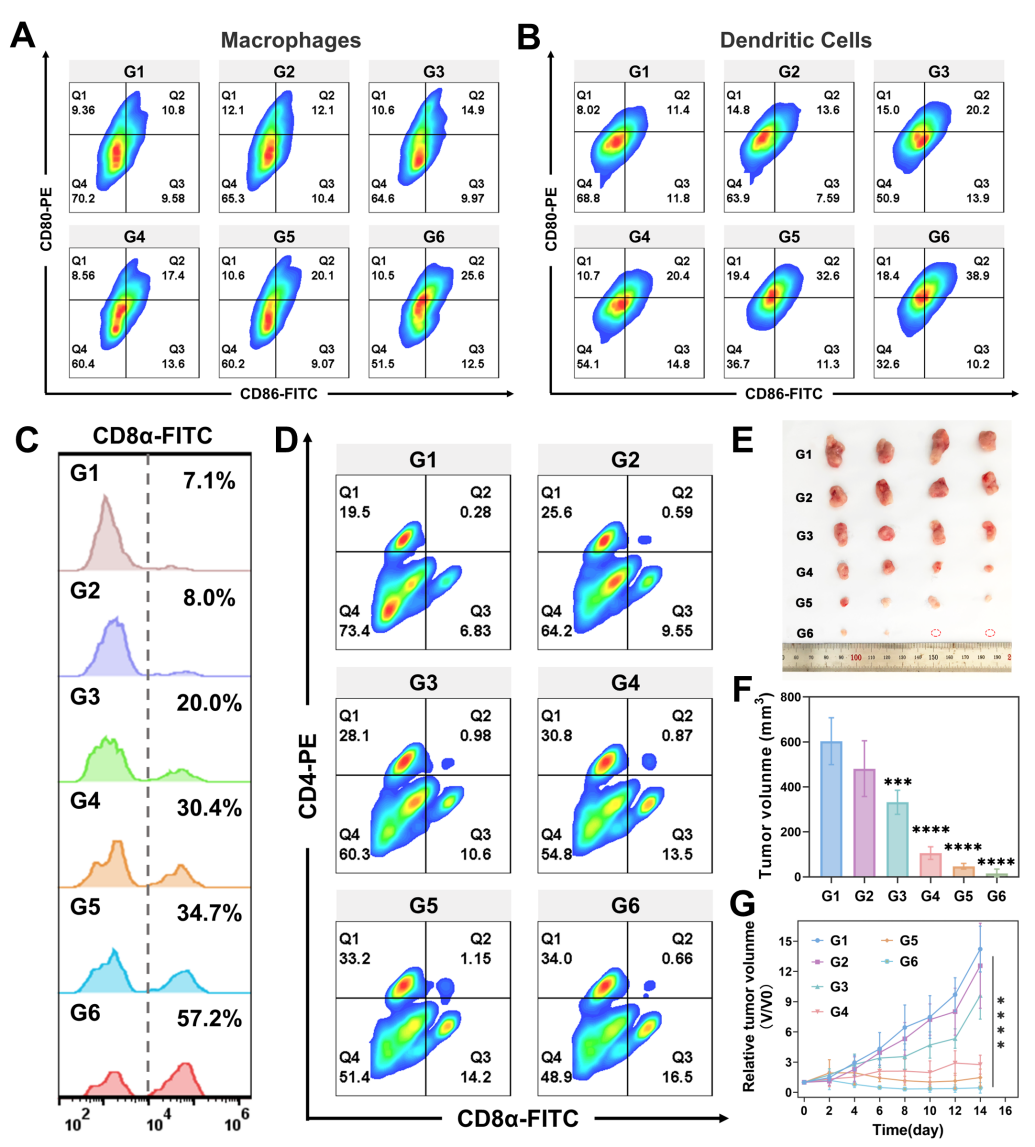
** GMCN@PEG enhances radiotherapy-induced immune response *in vivo*.** (A) Flow cytometry analysis of CD80^+^ and CD86^+^ Macrophages (gated on CD11b^+^ cells) in primary tumors after different treatments. G1: Control; G2: GNP@PEG; G3: GMCN@PEG; G4: IR; G5: IR+GNP@PEG; G6: IR+GMCN@PEG. (B) Flow cytometry analysis of CD80^+^ and CD86^+^ DCs (gated on CD11c^+^ cells) in primary tumors after different treatments. (C) Flow cytometry analysis of CD8^+^ T cells (gated on CD3^+^ cells) in primary tumors after different treatments. (D) Flow cytometry analysis of CD4^+^ and CD8^+^ T cells (gated on CD3^+^ T cells) in spleen tissues of 4T1 tumor-bearing mice with different treatments. (E) The image of the dissected distant tumors from each group on day 14. (F) Average tumor volume of distant tumors from each group on day 14. (G) Average tumor growth curves of distant tumors after various treatments. Statistical significance is indicated by asterisks: *p < 0.05, **p < 0.01, ***p < 0.001, ****p < 0.0001. Data are presented as means ± SD.

Our experiments *in vitro* cellular models have demonstrated that GMCN@PEG enhances radiotherapy-induced ICD, activates the cGAS-STING pathway, promotes DCs maturation, and facilitates M1 macrophages polarization, thereby enhancing antitumor immunity. To further validate these findings *in vivo*, flow cytometry analysis revealed that the IR+GMCN@PEG treatment group exhibited the highest proportion of mature DCs (CD11c^+^CD80^+^CD86^+^) and M1 macrophages (CD11b^+^CD80^+^CD86^+^), which are crucial for tumor antigen presentation to T cells and lead to the activation of T cell immune responses within the TME (Figure 8A-B). Consequently, we examined T cells infiltration and CD8^+^ T cells (cytotoxic T lymphocytes) levels in tumor tissues, as these are markers of antitumor immune activation 55. Flow cytometry data indicated a higher abundance of cytotoxic T lymphocytes (CD3^+^CD8^+^) in the tumors of mice treated with IR+GMCN@PEG compared to other treatment groups, a finding corroborated by immunofluorescence staining (Figure 8C, Figure S12D-E). The ELISA assays provided further evidence that levels of IFN-γ, a key regulator of the antitumor immune response, were significantly elevated in the tumors of the IR+GMCN@PEG treatment group compared to the other groups (Figure S12B) 56.

Furthermore, the combination of radiotherapy and GMCN@PEG treatment significantly increased splenic CD8^+^ and CD4^+^ T cell counts, indicating the activation of systemic immune responses (Figure 8D). Therefore, we investigated the efficacy of this therapeutic strategy against metastatic tumors. We developed 4T1 bilateral tumor mouse models by sequentially injecting 4T1 tumor cells into the right and left flanks of the mice, with the left flank serving as a model for distant metastasis (Figure S13A). During a 14-day observation period, we monitored tumor progression and changes in body weight in the mice (Figure 8E-F, Figure S13B). We observed that radiotherapy suppressed tumor growth, an effect that was significantly enhanced by the co-administration of GMCN@PEG, resulting in the complete eradication of certain metastatic lesions. Histological analysis using H&E and TUNEL staining corroborated these therapeutic effects (Figure S13E). Immunofluorescence and flow cytometry revealed a pronounced increase in CD8^+^ T cells within the metastatic lesions, particularly in the IR+GMCN@PEG group, signifying the activation of antitumor immunity at the metastatic sites (Figure S13E-F). A 40-day survival study demonstrated that the combined IR and GMCN@PEG treatment significantly prolonged the survival of mice with metastatic tumors compared to other treatment modalities (Figure S13C). The ELISA experiments further revealed that IFN-γ levels were highest in the tumors of the IR+GMCN@PEG treatment group compared to the other groups (Figure S13D). Collectively, these results suggest that GMCN@PEG enhances radiotherapy-induced antitumor immunity and activates systemic immunity to inhibit the growth of distal metastases.

To verify the effectiveness of GMCN@PEG in different tumor types, we evaluated its radiosensitizing effects in hepatocellular carcinoma (HCC) and colorectal cancer models. Results demonstrated that GMCN@PEG combined with radiotherapy significantly suppressed tumor growth (Figure S14A-D, Figure S15A-D). Mechanistically, this combination therapy markedly increased intratumoral ROS levels and enhanced cellular apoptosis (Figure S14E, Figure S15E). Furthermore, flow cytometry analysis revealed elevated CD8^+^ T cell infiltration in tumors treated with GMCN@PEG plus radiotherapy (Figure S14F, Figure S15F). These findings collectively indicate that GMCN@PEG potentiates radiotherapy across multiple cancer types and augments radiation-induced antitumor immunity.

## Conclusion

In summary, we have successfully developed an acidic TME responsive core-shell nanostructure, which serves as an innovative tool for enhanced MR-CT dual-modal imaging and radiotherapy. Our findings indicate that the GMCN@PEG exhibit high solubility and biocompatibility under neutral physiological conditions, while in the acidic TME, they demonstrate multi-enzymatic activities that ameliorate hypoxia and generate substantial ROS, thereby significantly enhancing the cytotoxic effects of radiotherapy on tumor cells. Moreover, GMCN@PEG facilitate the induction of ICD and activation of the cGAS-STING signaling pathway, thereby bolstering the antitumor immune response triggered by radiotherapy. *In vivo* studies in mice have shown that GMCN@PEG markedly enhance the suppressive effects of radiation on both primary and metastatic tumors, stimulate the activation of antitumor immunity, and notably extend the survival time of the animals. Additionally, GMCN@PEG exhibit superior performance in enhancing MR-CT imaging, offering the potential for integrated diagnosis and therapy. Collectively, GMCN@PEG, as a multifunctional nanosensitizer, has great potential in reshaping the tumor microenvironment, promoting radiotherapy, and enhancing biomedical imaging.

## Supplementary Material

Supplementary materials and methods, figures.
