# Supplementary material for: Nanosensitizer for enhanced radiotherapy via tumor microenvironment reshaping and ROS amplification
Source: Theranostics. 2026 Jan 1;16(3):1577–93. doi: 10.7150/thno.112311 (PMC12679703; doi:10.7150/thno.112311)
Supplement: Supplementary file 1 — Supplementary materials and methods, figures. [file thnov16p1577s1.pdf]

Supporting Information

Nanosensitizer for Enhanced Radiotherapy via Tumor Microenvironment Reshaping and ROS Amplification

Junchao Qian [1,2,3#], Yijun Lu [1,3#], Dandan Wang [3#], Shichuan Zhong [5], Xiao Liu [3], Xin Lai[3], Ziwen Wei [1,3], Guangyu Ju [3], Jinying Yang [1], Zimeng Wang[3], Kaiwei Wang[3], Yue Li [5,6*], Hongcang Gu [3*], Jian You [4*], Shuanghu Yuan [1,2*]

[1] Department of Radiation Oncology, The First Affiliated Hospital of USTC, Division of Life Sciences and Medicine, University of Science and Technology of China, Hefei, Anhui, 230031, P.R. China

[2] Department of Radiation Oncology, Anhui Provincial Cancer Hospital, Hefei, Anhui, 230031, P.R. China

[3] Anhui Province Key Laboratory of Medical Physics and Technology, Institute of Health and Medical Technology, Hefei Institutes of Physical Science, Chinese Academy of Sciences, Hefei 230031, P.R. China.

[4] College of Pharmaceutical Sciences, Zhejiang University, 866 Yuhangtang Road, Hangzhou, Zhejiang, 310058, P. R. China.

[5] Key Lab of Materials Physics, Anhui Key Lab of Nanomaterials and Nanotechnology, Institute of Solid State Physics, Hefei Institutes of Physical Science, Chinese Academy of Sciences, Hefei, Anhui, 230031, P. R. China.

[6] Tiangong University, Tianjin 300387, P. R. China.

Materials and Methods

Materials

Chloroauric acid tetrahydrate ($HAuCl_4 \cdot 4H_2O$, ≥47.8%), ethylene glycol ($C_2H_6O_2$, ≥99.5%), silver nitrate ($AgNO_3$, ≥99.8%), Citric acid monohydrate ($C_6H_8O_7 \cdot H_2O$, ≥99.8%), potassium permanganate ($KMnO_4$, ≥99.5%), potassium oxalate monohydrate ($C_2O_4K_2 \cdot H_2O$, ≥99.8%) and polyethylene glycol (PEG, Mw = 2000) were purchased from Sinopharm Chemical Reagent Co., Ltd (Shanghai, China). poly-(diallyldimethylammonium) chloride (PDDA, Mw = 100 000−200 000, 20 wt % in water) was purchased from Sigma-Aldrich. Hydrogen peroxide (H_2O_2, ≥30.0%), 3,3′,5,5′-tetramethylbenzidine (TMB, 99.0%), 5,5-dimethyl-1-pyrroline n-oxide (DMPO, 97%) were purchased from Aladdin Biochemical Technology Co., Ltd (Shanghai, China). The water used in the experiments is ultra-pure. All chemical reagents were used as received without further purification.

Synthesis of GNP

GNP was synthesized using a method previously reported[19]. Initially, 30 μL of 1 M $HAuCl_4$, followed by 1.2 mL of PDDA and 12 μL of 0.1 M $AgNO_3$ aqueous solution, were sequentially introduced into a glass vial containing 60 mL of EG. The mixture was then vigorously stirred at room temperature to ensure a homogeneous distribution. Subsequently, the prepared solution was heated in an oil bath at 220 °C for 2 hours, during which the color of the solution transitioned from yellow to red. The resulting nanoparticles were isolated by centrifugation at 10,000 rpm and subjected to repeated washing with ultra-pure water, followed by ultrasonication, to eliminate remaining EG and PDDA. The volume of the nanoparticle solution was then adjusted to 5 mL.

Synthesis of GMCN

The prepared GNP solution was incubated with 25 mL of a 10 mM citric acid (CA) solution for 12 hours. Following the incubation, the CA-modified GNP was isolated by centrifugation at 10,000 rpm, and then washed thoroughly with ultra-pure water and

subjected to ultrasonication to eliminate excess citric acid. The volume of the GNP solution was adjusted to 30 mL. Subsequently, 0.8 mL of 0.1 M $KMnO_4$ and 4 mL of 0.1 M potassium oxalate $K_2C_2O_4$ were added to the solution. The mixture was sonicated for 1 minute to ensure a uniform distribution. The solution was then heated in a water bath at 60 °C for 2 hours. Upon completion of the reaction, the product was collected by centrifugation at 8000 rpm, and residual $KMnO_4$ and $K_2C_2O_4$ were removed by repeated washing with ultra-pure water and ultrasonication. The volume of the nanoparticle solution was then adjusted to 5 mL.

PEG modification of GMCN and GNP

The GNP/GMCN solution prepared previously was combined with 150 mg of PEG 2000 and added to 50 mL of ultra-pure water. The mixture was sonicated for 30 minutes to ensure a uniform dispersion, followed by stirring with a magnetic stirrer for 6 hours to promote interaction between the components. After the stirring period, the product was collected by centrifugation at 10,000 rpm, and the supernatant containing excess PEG was removed by repeated washing with ultra-pure water and ultrasonication.

Characterization of the GMCN

The morphology of the final product was characterized by field emission scanning electron microscopy (SEM, ZEISS, ΣIGMA). The products were characterized by transmission electron microscopy (TEM, JEOL, JEM-1400). For TEM analysis, a droplet of the prepared solution was deposited onto a carbon-coated copper grid, followed by evaporation at ambient temperature. The EDS mapping and HAADF-STEM were carried out using Talos F200X and JEOL Arm-200F electron microscopes. X-ray photoelectron spectroscopy (XPS) was conducted on an ESCALAB 250 X-ray photoelectron spectrometer, employing Al Kα radiation. The UV-visible (UV-vis) absorbance spectra were recorded using a UV-1601 spectrophotometer. The molar and mass ratios of Au and manganese Mn in GMCN were determined using inductively coupled plasma mass spectrometry (ICP-MS, Thermo, icap Qc). Furthermore, the zeta

potential, hydrodynamic diameter, and polydispersity index (PDI) of the samples were determined using a Malvern Zetasizer Nano ZS90.

Measurement of POD-like activity

The concentration dependence of the POD-like activity of GMCN was measured by adding different concentrations of GMCN (0, 5, 10, 20, and 40 μg mL^{-1}) and 1 mM TMB in H_2O_2 solution (100 mM) at pH 6.0. The final 652 nm absorbance of the mixture was detected by a UV-vis spectrophotometer. To study the effect of pH values on the POD-like activity of GMCN, 40 μg mL^{-1} GMCN and 1 mM TMB were added in H_2O_2 (100 mM) at different pH values. The absorbance was measured as same as described above. The steady kinetic assay of GMCN using H_2O_2 as substrate was performed by adding 40 μg mL^{-1} GMCN and 1mM TMB in different concentrations of H_2O_2 solution (50, 100, 150, 200, 250, and 300 mM). Then, the absorbance values obtained at 652 nm were converted to the concentration of TMB-derived oxidation products by the Beer-Lambert Law. The Michaelis–Menten constant was determined from the Michaelis-Menten saturation curve.

GMCN generation · OH radical detection

Take a capillary tube of 20 μL of water or the GMCN solution and put it into the electron spin resonance (ESR) device (Bruker EMX plus 10/20). Hydroxyl radical spin adduct (DMPO/·OH) was used to detect the characteristic peak (1:2:2:1) of the GMCN catalyzed H_2O_2 to ·OH at different pH values. The content of ·OH was quantitatively estimated by the ESR signal intensity.

Measurement of CAT-like activity

The concentration dependence of the CAT-like activity of GMCN was measured by adding different concentrations of GMCN (0, 5, 10, 20, and 30 μg mL^{-1}) in H_2O_2 solution (200 mM) at pH 6.0. The generated O_2 solubility was measured after different reaction times. The steady-kinetic assays of GMCN using H_2O_2 as substrate were

performed by adding 20 μg mL^{-1} GMCN in different concentrations of H_2O_2 solution (50, 100, 150, 200, 250, and 300 mM). The generated O_2 solubility was monitored at different reaction times. The Michaelis-Menten constant was determined from the Michaelis–Menten saturation curve.

In vitro cell viability

To evaluate cell viability, 4T1 cells were plated in 96-well plates at a density of 5,000 cells per well and incubated for 24 hours. The culture medium was then aspirated from each well, and the cells were exposed to a range of GNP@PEG/GMCN@PEG concentrations (0, 12.5, 25, 50, 100 μg mL^{-1}) in either pH 6.0 or pH 7.4 medium for 8 hours, after which they were subjected to X-ray irradiation at a dose of 10 Gy. The cells were further incubated for 24 hours post-irradiation. Subsequently, the medium was replaced with 100 μL of fresh medium containing the CCK-8 reagent, and the cells were incubated for an additional 2 hours. The optical density (OD) at 450 nm was subsequently measured using a spectrophotometer.

Calcein-AM/PI live/dead cell double staining

Live/dead cell double staining was performed using a Calcein-AM/PI staining kit. 4T1 cells were seeded into 12-well plates at a density of 100,000 cells per well and incubated for 12 hours. The cells were then treated with GNP@PEG/GMCN@PEG at a concentration of 100 μg mL^{-1} in either pH 6.0 or pH 7.4 medium for 8 hours. Following treatment, they were subjected to X-ray irradiation at a dose of 10 Gy and further incubated for 24 hours. After the post-irradiation incubation, the culture medium was aspirated, and the cells were washed twice with PBS. Subsequently, the cells were stained with a solution containing 2 μM Calcein-AM and 4.5 μM PI and incubated for 30 minutes. After staining, the cells were washed three times with PBS and visualized under a fluorescence microscope.

Colony formation assay

To evaluate the sensitizing effect of radiotherapy in GMCN@PEG, colony formation assays were conducted. 4T1 cells were seeded in 6-well plates at a density of 1,000 cells per well and cultured for 12 hours. Following this initial incubation, the cells were treated with 50 μg mL^{-1} of GNP@PEG/GMCN@PEG for 2 hours, after which they were exposed to X-ray irradiation. The cells were then cultured for 14 days, with the culture medium being refreshed every 3 to 4 days. At the end of the incubation period, the cells were fixed using 4% paraformaldehyde (PFA) and stained with 10% crystal violet dye. Following cell enumeration with ImageJ, the sensitization to radiotherapy was quantified through the calculation of the survival fraction (SF) and the dose modification factor (DMF).

DNA double-stranded break detection

4T1 cells were seeded into 12-well plates at a density of 100,000 cells per well. After a 24-hour incubation, the cells underwent the same treatment process as previously described. Following this, the cells were fixed with 4% paraformaldehyde for 1 hour. Subsequently, 5% bovine serum albumin (BSA) was applied to block the samples for 1 hour at 37 °C. The samples were then co-incubated with an anti-γ-H2AX antibody diluted at 1:1000 and a FITC-labeled goat anti-mouse IgG secondary antibody, also diluted at 1:1000. The final observation was conducted using fluorescence microscopy.

Evaluation of intracellular O_2 and ROS generation

Intracellular O_2 generation was assessed using the $[Ru(dpp)_3]Cl_2$ fluorescent probe. 4T1 cells were seeded into 12-well plates at a density of 100,000 cells per well. After a 24-hour incubation, the cells underwent the same treatment process as previously described. Subsequently, the cells were stained with a solution containing 30 μM $[Ru(dpp)_3]Cl_2$ and incubated for 30 minutes. After staining, the cells were washed three times with PBS and visualized under a fluorescence microscope.

Intracellular reactive oxygen species (ROS) production was assessed using the DCFH-DA fluorescent probe, following the same procedure as previously described for the

detection of O_2.

Calreticulin detection

4T1 cells were seeded into 12-well plates at a density of 100,000 cells per well. After a 24-hour incubation, the cells underwent the same treatment process as previously described. Following this, the cells were fixed with 4% paraformaldehyde for 1 hour. Subsequently, 5% bovine serum albumin (BSA) was applied to block the samples for 1 hour at 37°C. The samples were then co-incubated with an anti- Calreticulin primary antibody (Bioss, bs-25628R) diluted at 1:1000 and a FITC-labeled goat anti-rabbit IgG secondary antibody, also diluted at 1:1000. The final observation was conducted using fluorescence microscopy.

***In vitro* cytokine detection**

Cells in the logarithmic growth phase were seeded into 6-well plates at a density of 50,000 cells per well. After a 24-hour incubation, the cells were subjected to different treatments. Following the treatments, the cells were further incubated for an additional 24 hours. Subsequently, the supernatant was collected from each well. The cytokines present in the supernatant were quantified using a specific enzyme-linked immunosorbent assay (ELISA) kit. The optical density (OD) at 450 nm was subsequently measured using a spectrophotometer.

Western blot analysis

Total protein was extracted from tumor tissues using RIPA lysis buffer supplemented with 1× protease/phosphatase inhibitor cocktail. Protein concentrations were determined by BCA assay. Samples (30 μg/lane) were separated by 10% SDS-PAGE and transferred to PVDF membranes. After blocking with 5% BSA/TBST for 1 h, membranes were incubated overnight at 4 °C with primary antibodies against: STING (Proteintech, 19851-1-AP); p-STING (Ser366) (Affinity Biosciences, AF7416); TBK1 (Affinity Biosciences, DF7026); p-TBK1 (Ser172) (Affinity Biosciences, AF8190);

IRF3 (Affinity Biosciences, DF6895); p-IRF3 (Ser396) (Affinity Biosciences, AF2436). Following TBST washes, membranes were incubated with HRP-conjugated secondary antibodies for 1 h at room temperature. Protein bands were visualized using ECL substrate and quantified by ImageJ software.

In vitro and *in vivo* MR and CT imaging

In vitro MRI measurements were conducted using a Philips Achieva 3.0T MRI scanner with a head coil. For *in vivo* MRI, a Philips Achieva 3.0T MRI scanner equipped with an animal coil was utilized. GMCN@PEG at varying concentrations (0, 0.0125, 0.025, 0.05, 0.1 mM of [Mn]) were adjusted to pH levels of 7.4 and 6.0, respectively, and the T1 signal intensity of these solutions was measured. For *in vivo* MRI experiments, T1-weighted images were acquired following the intravenous injection of GMCN@PEG (200 μL at a dosage of 10 mg kg^{-1}) into mice. Subsequently, T1-weighted MR images were captured at time points of 0, 0.5, 1, 2, and 24 hours post-injection. The mean MR gray value at the tumor site was quantified using ImageJ software.

In vitro and *in vivo* computed tomography (CT) measurements were performed using a Philips Brilliance CT scanner. The experimental procedures, excluding the imaging modality, were consistent with those previously described for magnetic resonance (MR) imaging.

Hematological analysis

Mice were intravenously injected with either PBS as a control or a solution of GMCN@PEG at a concentration of 200 μL of 1 mg mL^{-1}. Blood routine and biochemical indexes were quantitatively analyzed at various time points post-injection: 24 hours, 72 hours, and 120 hours. For blood biochemical analysis, 200 μL of blood from each mouse was collected in tubes containing 0.15% (mass/volume) ethylenediaminetetraacetic acid potassium salt dihydrate ($EDTA\text{-}K_2{\cdot}2H_2O$). The blood samples were then centrifuged at 3500 revolutions per minute (rpm) for 8 minutes at 4 °C and the supernatant was collected for analysis.

Hematoxylin and Eosin (H&E) staining

The main organs (heart, liver, spleen, lung, and kidney) of the mice were collected and fixed overnight in 4% PFA. The tissue was then dehydrated in 30% sucrose and cut into 20-μm slices for staining. The nucleus was stained with 1% eosin (Biosharp, BL702B) for 10 minutes, and the cytoplasm was stained with hematoxylin (Biosharp, BL703B) for 2 minutes. To selectively remove excess dye, the slices were dipped in 1% acid alcohol for a few seconds and then sealed with a neutral resin.

Evaluation of biodistribution *in vivo*

The main organs (heart, liver, spleen, lung, and kidney) and tumors from mice treated with GMCN@PEG were harvested at 24, 72, and 120 hours post-injection. These organs and tumors were then dissolved in chloroazotic acid and incubated overnight. Following incubation, all samples were centrifuged at 12,000 revolutions per minute (rpm) for 20 minutes and diluted 100-fold to measure the gold concentration using inductively coupled plasma (ICP) analysis.

The half-life of *in vivo* circulation of GMCN@PEG

To investigate the half-life of *in vivo* circulation of GMCN@PEG, all mice were intravenously injected with the solution (200 μL at a dosage of 10 mg kg^{-1}). Blood samples (10 μL) were collected at various time points post-injection: 2, 8, 15, 30 minutes, and 1, 2, 4, 12, 24, and 48 hours. Each blood sample was mixed with 10 μL of an anticoagulant solution containing 0.15% (mass/volume) EDTA-K2·$2H_2O$. The blood samples were dissolved in nitric acid overnight and then diluted 100 times. The gold content was estimated using inductively coupled plasma optical emission spectrometry (ICP-OES). The circulating half-life (τ1/2) of GMCN@PEG in blood was determined using a two-compartment pharmacokinetic model. The elimination rate curve was plotted by fitting the natural logarithm of the plasma concentration (ln(Cp)) against time. The slope of this curve represented the elimination rate constant.

Animal experiments

Female BALB/c mice, aged 4-5 weeks, were sourced from the Animal Laboratory Center of Anhui Medical University in Hefei, China. The study protocol, along with all procedures related to animal care and treatment, received approval from the Animal Care and Use Committee of the Hefei Institute of Physical Sciences, Chinese Academy of Sciences.

A primary 4T1 tumor mouse model was established via subcutaneous injection of a 100 μL 4T1 cell suspension (1×10^6 cells mL^{-1}) into the right hind limb of each BALB/c mouse. Mice with primary tumors measuring 70 to 100 mm^3 were selected for *in vivo* experiments.

A metastatic 4T1 tumor mouse model was similarly established. Initially, a 100 μL 4T1 cell suspension (1×10^6 cells mL^{-1}) was injected subcutaneously into the right hind limb of each BALB/c mouse. After three days, a second subcutaneous injection of the same concentration of 4T1 cell suspension (100 μL) was administered into the back of the left hind limb to induce a distal metastatic tumor. Mice bearing primary tumors within the size range of 70 to 100 mm^3 were utilized for *in vivo* experiments.

A hepatocellular carcinoma or colorectal cancer mouse model was established via subcutaneous injection of a 100 μL Hepa1-6 or CT26 cell suspension (1×10^6 cells mL^{-1}) into the right hind limb of each BALB/c mouse. Mice with tumors measuring 70 to 100 mm^3 were selected for *in vivo* experiments.

***In vivo* antitumor study**

To explore the impact of GMCN@PEG on enhancing radiotherapy sensitivity and stimulating an immune response in primary tumors, BALB/c mice bearing primary subcutaneous 4T1 tumors were randomly assigned to six experimental groups, each consisting of 10 mice: G1: Control; G2: GNP@PEG; G3: GMCN@PEG; G4: IR; G5: IR+GNP@PEG; G6: IR+GMCN@PEG. GNP@PEG, GMCN@PEG, or PBS were injected directly into the tumor tissue. 12 hours post-injection, groups 4-6 underwent

X-ray irradiation at the tumor sites. Three treatments in total,the treatment initiation was marked as Day 0, and the mice were weighed at one-day intervals. Tumor volume was measured using the formula: $V = L \times W^2 / 2$, where V represents the tumor volume, L is the tumor length, and W is the tumor width.

To further investigate the influence of GMCN@PEG on the enhancement of radiotherapy sensitivity and immune response in metastatic tumors, BALB/c mice with metastatic subcutaneous tumors were randomly assigned to six experimental groups, each consisting of 10 mice. Direct injections of PBS, GNP, or GMCN@PEG were administered into the tumor tissue. 12 hours post-injection, groups 4-6 received X-ray irradiation at the primary tumor sites. The body weight and survival status of the mice were monitored daily. Mice were considered to have reached the end-point of the study when the volume of the right-sided tumor reached 1500 mm^3. The dimensions of the bilateral tumors were measured and recorded on alternate days for a period of 14 days.

H&E staining of tumor

Upon completion of the treatment cycle, the mice were humanely euthanized. The tumor tissues were carefully dissected and subsequently fixed in a 10% paraformaldehyde solution, fixed in 10% paraformaldehyde, paraffin-embedded, then sectioned (4 μm thickness). The slices were stained with H&E and imaged using an optical microscope microscope.

ROS detection of tumor

Following the conclusion of the treatment cycle, the mice were humanely euthanized. The tumor tissues were promptly dissected and embedded using Optimal Cutting Temperature (OTC) compound to preserve cellular structures for cryosectioning. The frozen sections were then prepared and immediately stained with the DCFH-DA fluorescent probe to detect ROS. Finally, the sections were counterstained with 4',6-diamidino-2-phenylindole (DAPI), a fluorescent stain for nuclear DNA, and examined using a fluorescence microscope to capture images of the tissue.

TUNEL staining of tumor

Tumor tissue samples were collected and fixed in a 4% paraformaldehyde solution to preserve cellular structures. Following fixation, the tissues underwent a dehydration process and were subsequently embedded in paraffin. Thin sections were cut from the embedded tissue for further analysis. The tumor tissue sections were then incubated with terminal deoxynucleotidyl transferase (TdT) enzyme at 37 °C for one hour to facilitate the detection of DNA breaks. After incubation with TdT, the sections were thoroughly cleaned and incubated with a streptavidin-tetramethylrhodamine (TRITC) labeling solution at 37 °C for 30 minutes to enhance the visualization of the target. Finally, the sections were counterstained with DAPI, a fluorescent stain for nuclear DNA, and examined using a fluorescence microscope to capture images of the tissue.

Hypoxia detection of tumor

Tumor hypoxia was assessed using the Hypoxyprobe™-1 Kit (Hypoxyprobe, HP1-100Kit, USA). Mice received intraperitoneal injection of pimonidazole HCl (60 mg/kg) 90 min prior to sacrifice. Excised tumors were fixed in 4% paraformaldehyde for 24 h, paraffin-embedded, and sectioned at 5 μm thickness. After deparaffinization and antigen retrieval (citrate buffer, pH 6.0, 95 °C, 20 min), sections were blocked with 5% BSA and incubated with FITC-conjugated anti-pimonidazole monoclonal antibody (1:50 dilution) overnight at 4 °C. Nuclei were counterstained with DAPI. Fluorescence images were acquired using a fluorescence microscope with consistent exposure settings across all samples.

Immunofluorescence staining of tumor

Tumor hypoxia improvement, indicated by the expression of Hypoxia-Inducible Factor 1-alpha (HIF1α), and tumor immune enhancement, as measured by $CD8^+$ T cell infiltration, were assessed using immunofluorescence staining. Following treatment, tumor tissues were excised via dissection and fixed in a 4% paraformaldehyde solution

to preserve cellular structures. The fixed tissues were then dehydrated, embedded in paraffin, and sectioned for further analysis. The immunofluorescence staining was conducted using specific primary antibodies: rabbit monoclonal anti-HIF1α (Abcam, catalog number ab28459) and mouse monoclonal anti-CD8 (Abcam, catalog number ab56847). Subsequently, the sections were incubated with the corresponding secondary antibodies: Alexa Fluor 549 (AF549, Abcam, catalog number ab63485) and Alexa Fluor 488 (AF488, Abcam, catalog number ab63528). Finally, the stained sections were examined and photographed using a fluorescence microscope to visualize the fluorescence signals.

Flow Cytometry of tumor

Tumor and spleen tissues from different groups were minced with scissors and digested with collagenase. Using a 70-μm cell filter, the dissociated cells were filtered into a 50 mL centrifuge tube containing 5 mL erythrocyte lysate to obtain a single cell suspension. Following incubation for 5 min at 37 °C, 5 mL complete DMEM was used to terminate the reaction. Centrifugation at 1200 rpm for 5 min was followed by three washings in PBS and resuspension in complete DMEM for 10 mL. Subsequently, different cell populations in the cell suspension were stained using specific antibodies. DCs were labeled with anti-CD11b-APC (BioLegend, 101212), anti-CD80-PE (BioLegend, 104708), and anti-CD86-APC (BioLegend, 105006) antibodies. M1-type macrophages were identified and labeled with anti-CD11c-APC (BioLegend, 117310), anti-CD80-PE, and anti-CD86-APC antibodies. Cytotoxic T cells were labeled with anti-CD3-APC (BioLegend, 100236), anti-CD4-PE (BioLegend, 100408), and anti-CD8-Alexa Fluor 488 (BioLegend, 100723) antibodies. After a 30-minute staining period, the distinct cell populations were analyzed using flow cytometry.

Statistical analysis

All graphs and statistical analysis were performed using GraphPad Prism 9.3 and OriginLab Origin 2022 software. The unpaired t-test was used to compare differences

between two groups, while the one-way ANOVA was used for differences between multiple groups. The values obtained from the analysis were expressed as mean ± standard deviation (SD). A significance level of $P<0.05$ was considered statistically significant.

A

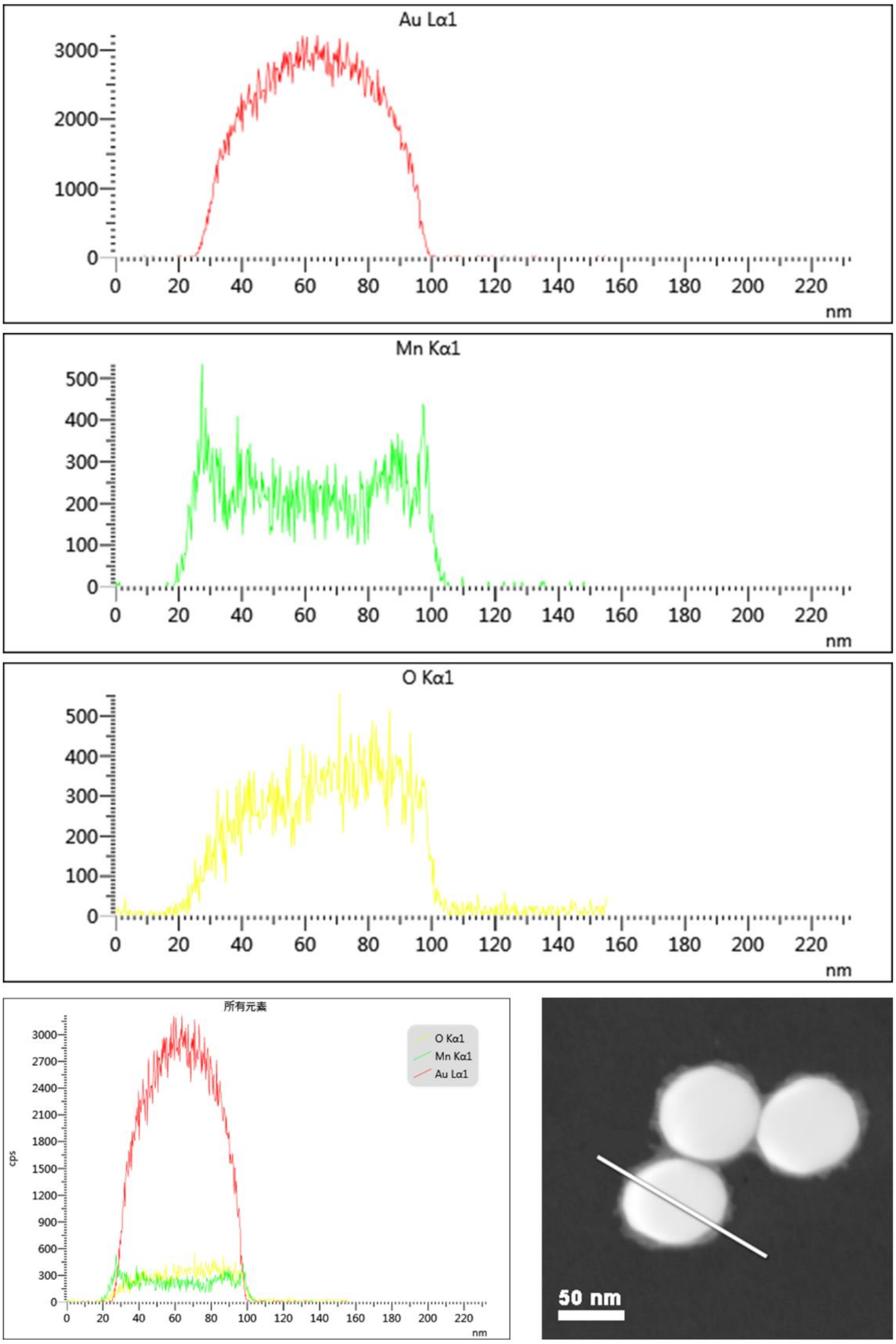

Figure S1. Elemental composition and distribution of GMCN. (A) EDS line scan analysis of GMCN.

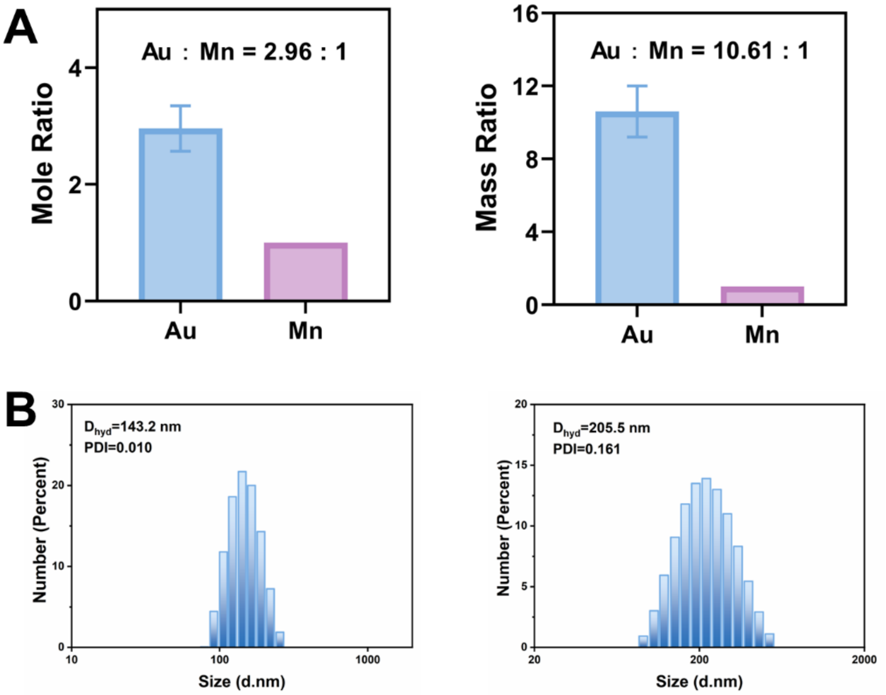

Figure S2. Elemental composition and particle size of GMCN. (A) The molar and mass ratios of the elements Au and Mn. Left panel: mole ratio, right panel: mass ratio. (B) Particle size of GNP and GMCN. Left panel: GNP, right panel: GMCN.

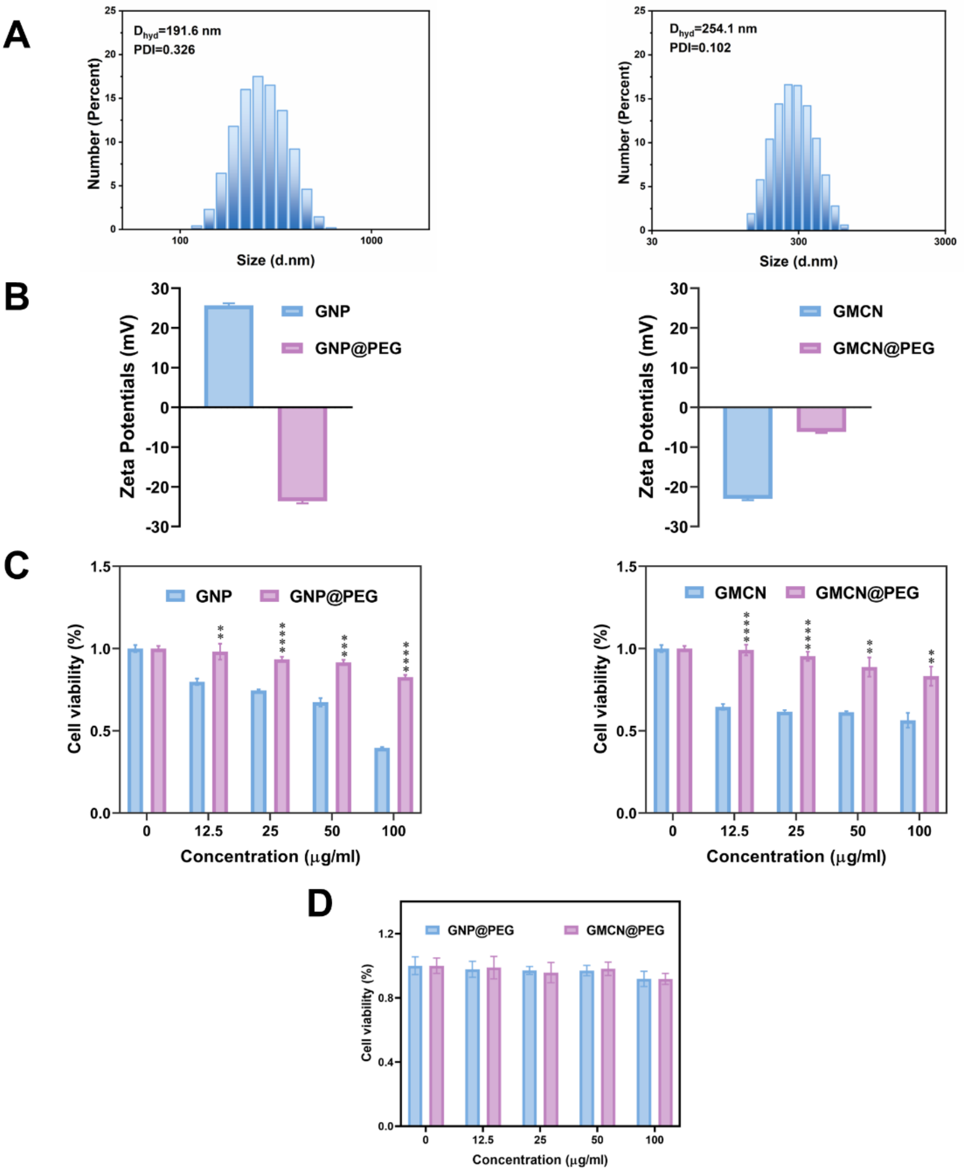

Figure S3. Comparative of GNP and GMCN before and after PEGylation. (A) Particle size of the PEGylated GNP and GMCN. Left panel: GNP, right panel: GMCN. (B) Comparative Zeta potential of GNP and GMCN before and after PEGylation. Left panel: GNP, right panel: GMCN. (C) Comparative cytotoxicity evaluation of GNP and GMCN before and after PEGylation in 4T1 cells (pH 7.4). Left panel: GNP, right panel: GMCN. (D) Viability of HEK293 cells (human embryonic kidney cells) treated with different concentrations of GNP@PEG and GMCN@PEG. Statistical significance is indicated by asterisks: *$p < 0.05$, **$p < 0.01$, ***$p < 0.001$, ****$p < 0.0001$. Data are presented as means ± SD.

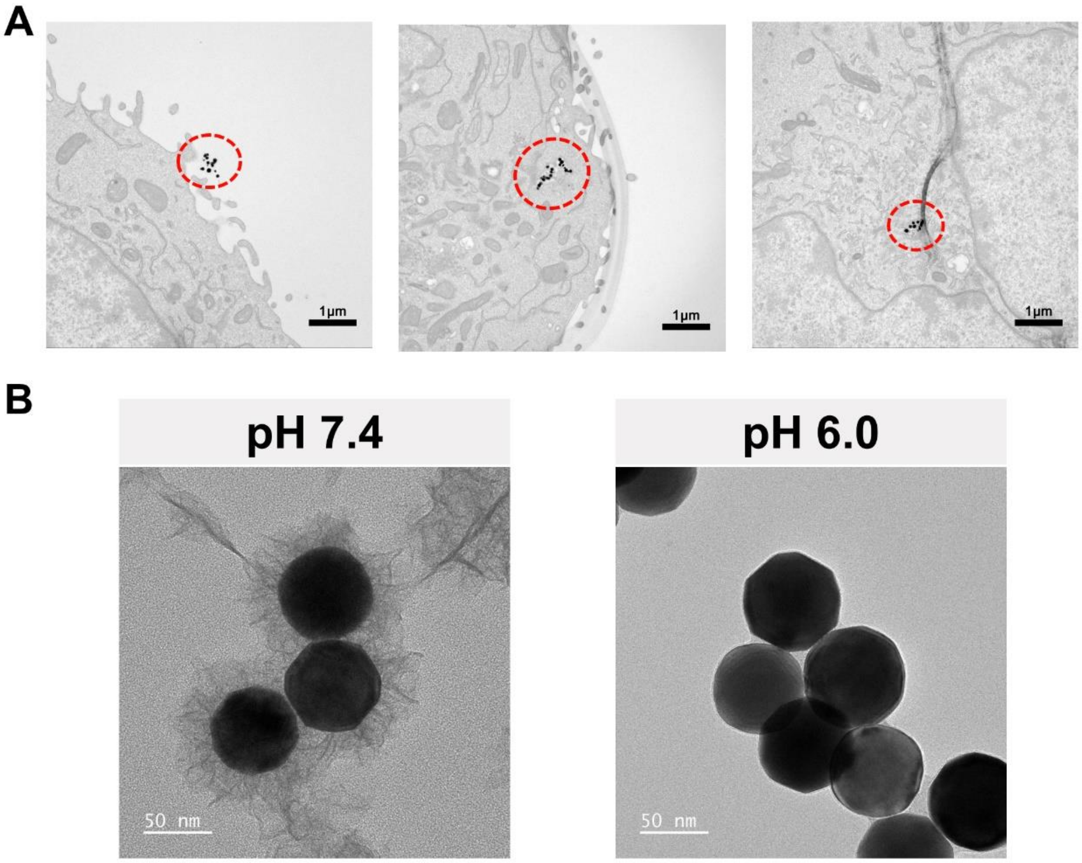

Figure S4. Cellular uptake and acid-responsive degradation of GMCN@PEG. (A) Bio-TEM images of 4T1 cells incubated with the GMCN@PEG. (B) TEM images of GMCN@PEG after 6 h incubation at pH 7.4 and pH 6.0.

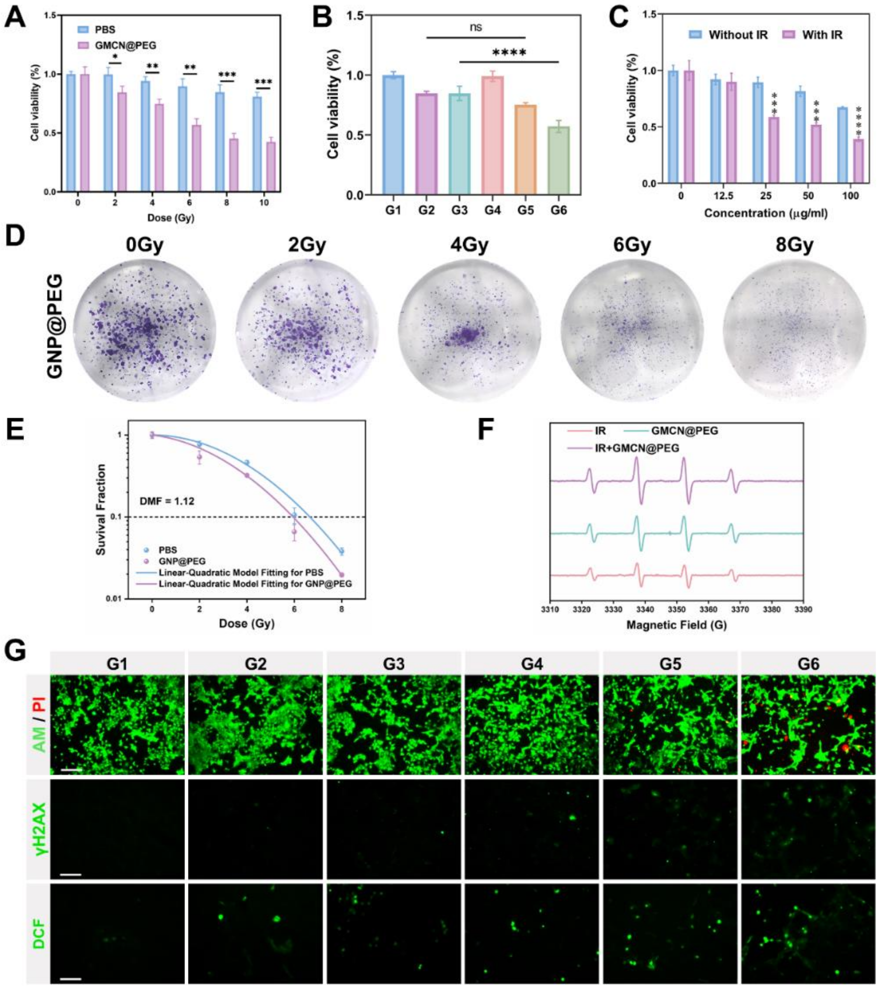

Figure S5. Radiotherapy sensitization of GMCN@PEG *in vitro*. (A) Viability of 4T1 cells treat with PBS and GMCN@PEG under various X-ray doses. (B) Viability of 4T1 cells with different treated groups at pH 7.4. G1: Control; G2: GNP@PEG; G3: GMCN@PEG; G4: IR; G5: IR+GNP@PEG; G6: IR+GMCN@PEG. (C) Viability of 4T1 cells treated with different concentrations of GNP@PEG, with or without IR exposure. (D) Representative colonies of 4T1 cells treated with GNP@PEG under various X-ray doses. (E) Clonogenic survival curves of 4T1 cells treat with PBS and GNP@PEG under various X-ray doses. (F) ESR spectra for •OH at different conditions (GMCN@PEG=40 μg mL^{-1}, H_2O_2=100 mM, pH=6.0, X-ray dose=8 Gy). (G) Confocal fluorescence microscopy images of 4T1 cells stained for calcein-AM/PI, γH2AX, and DCFH-DA, representing different treatment groups at pH 7.4. Scale bar: 100 μm. Statistical significance is indicated by asterisks: *$p < 0.05$, **$p < 0.01$, ***$p < 0.001$, ****$p < 0.0001$. Data are presented as means ± SD.

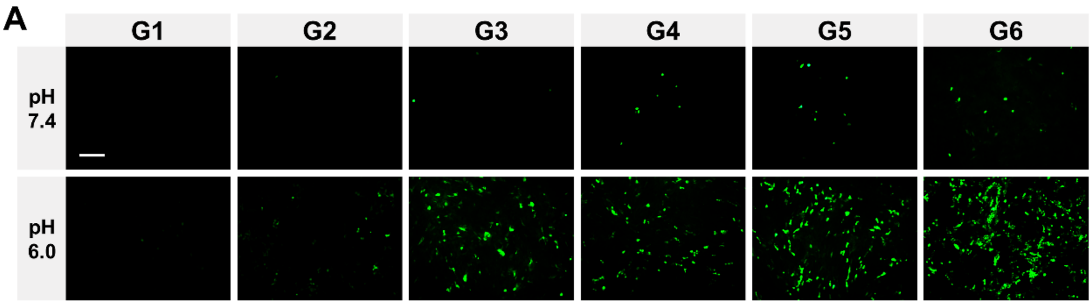

Figure S6. (A) Confocal fluorescence microscopy images of DCFH-DA staining in 4T1 cells across treatment groups after endogenous oxidases depletion. Scale bar: 100 μm.

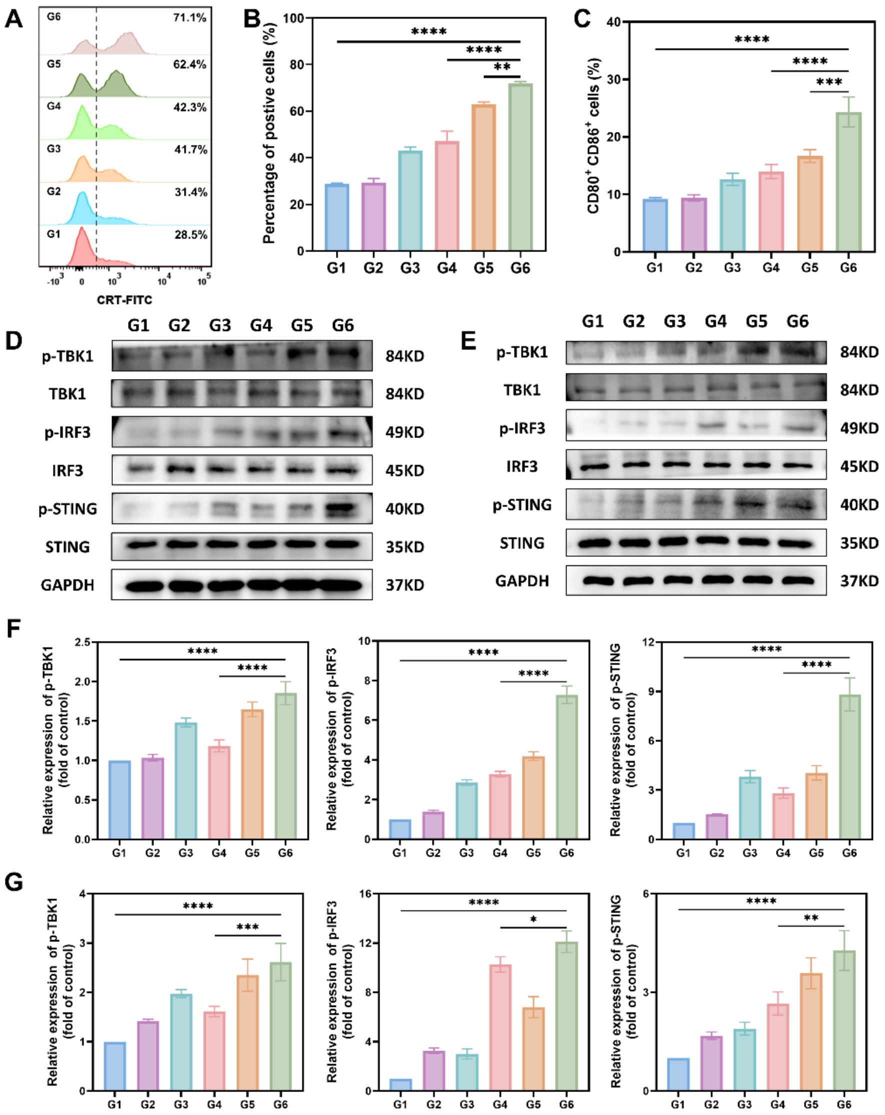

Figure S7. GMCN@PEG enhances radiotherapy-induced immune response *in vitro*. (A) Flow cytometry analysis of surface CRT levels in 4T1 cells under different treatments. G1: Control; G2: GNP@PEG; G3: GMCN@PEG; G4: IR; G5: IR+GNP@PEG; G6: IR+GMCN@PEG. (B) Flow cytometric quantification of surface CRT levels in 4T1 under different treatments. (C) Flow cytometric quantification of $CD80^{+}CD86^{+}$ cells in DC under different treatments. (D, E) Western blot analysis of the STING pathway-relative protein expression in DCs (D) and macrophages (E) after treatment with various conditions. (F, G) Quantification of STING pathway-relative proteins in DCs (F) and macrophages (G) by Western blot across treatment groups. Statistical significance is indicated by asterisks: *p < 0.05, **p < 0.01, ***p < 0.001, ****p < 0.0001. Data are presented as means ± SD.

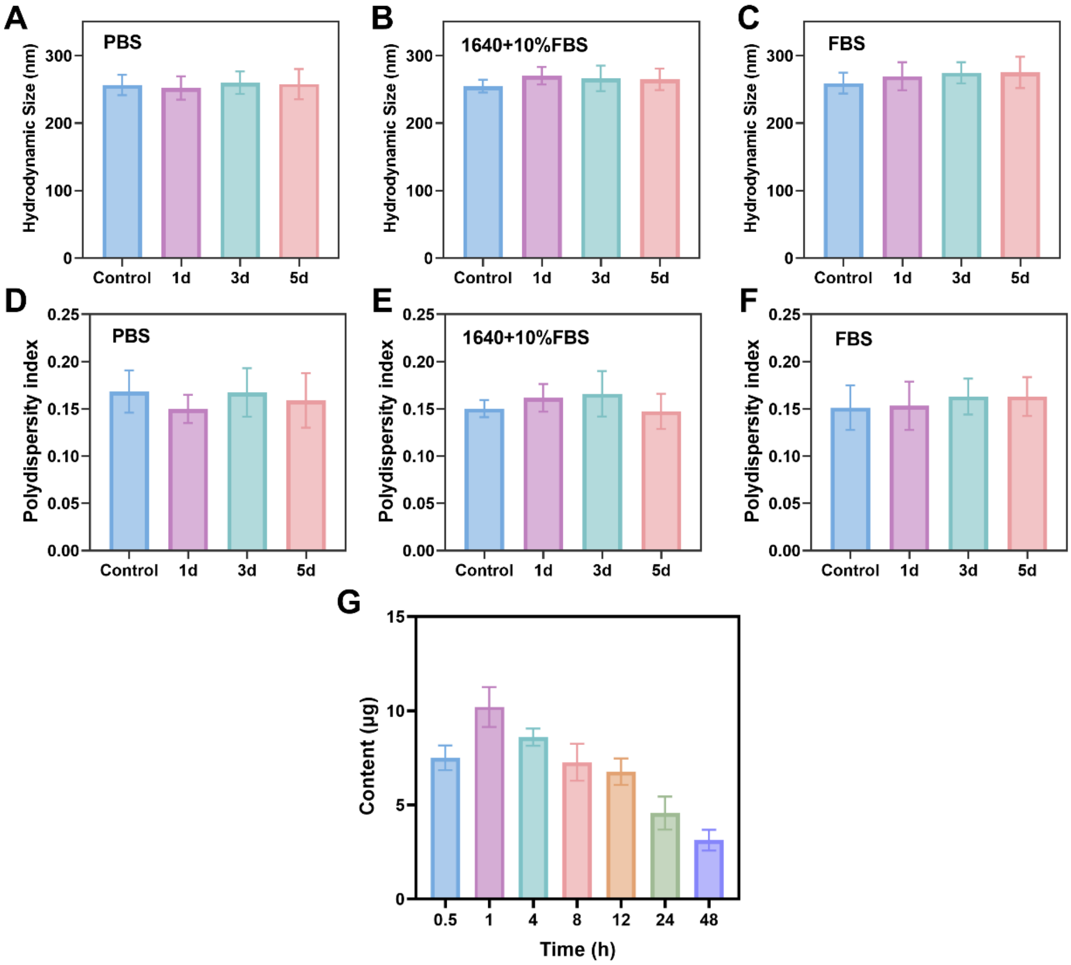

Figure S8. Biological safety and biocompatibility of GMCN@PEG *in vivo*. (A-C) Hydrodynamic diameter changes of GMCN@PEG in PBS (A), RPMI-1640 + 10% FBS (B), and 100% FBS (C) after 1, 3, and 5 days of incubation. (D-F) PDI changes of GMCN@PEG in PBS (D), RPMI-1640 + 10% FBS (E), and 100% FBS (F) after 1, 3, and 5 days of incubation. (G) GMCN@PEG accumulation in tumors at different time post-intravenous injection.

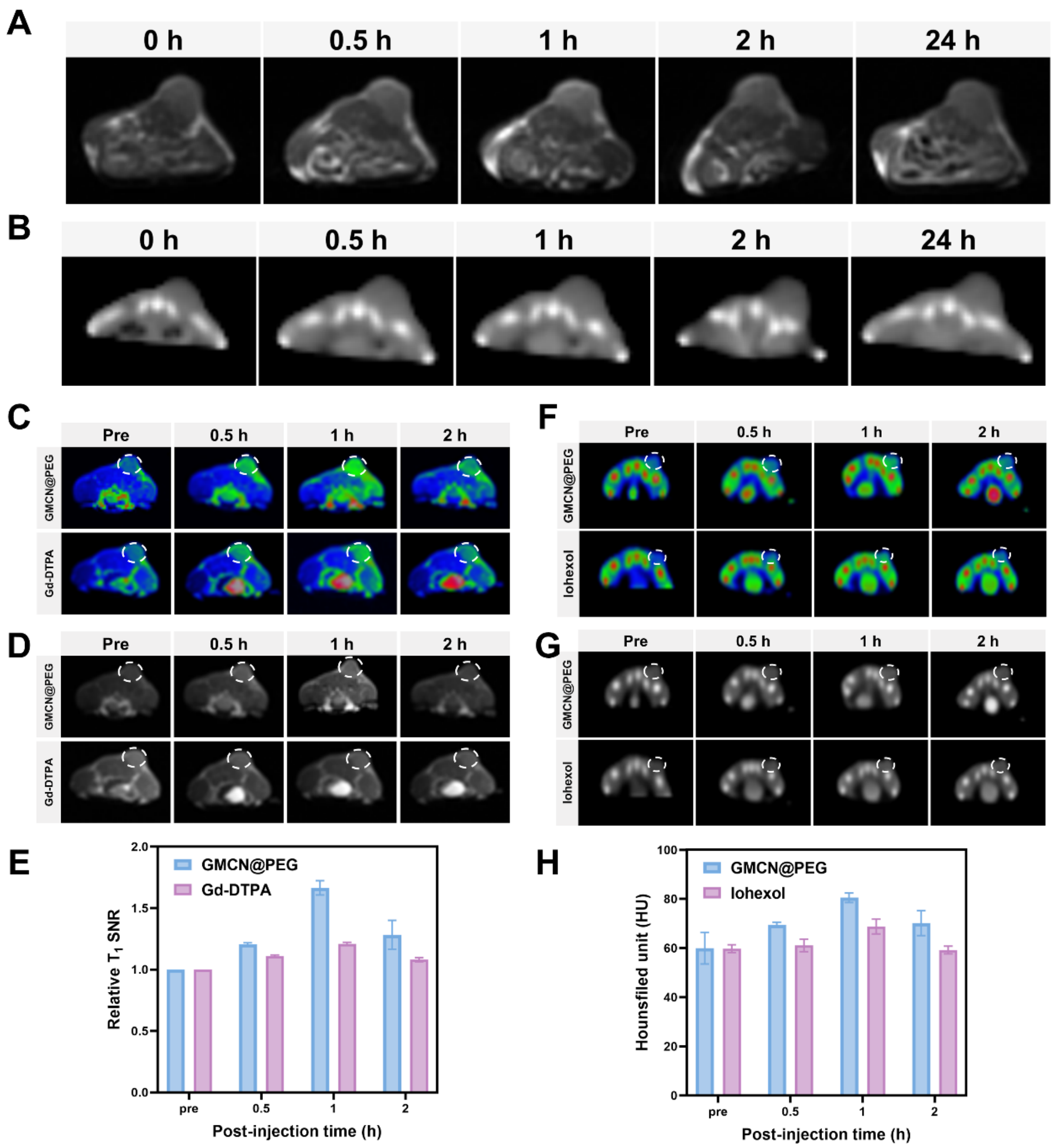

Figure S9. MR-CT dual-mode imaging enhancement of GMCN@PEG *in vivo*. (A) T_1-weighted MR grayscale images of 4T1 tumor bearing BALB/c mice after i.v. injection of GMCN@PEG (10 mg kg^{-1}) at various time points. (B) CT grayscale images of 4T1 tumor bearing BALB/c mice after i.v. injection of GMCN@PEG (10 mg kg^{-1}) at various time points. (C, D) T1-weighted MRI of 4T1 tumor bearing BALB/c mice after i.v. injection of GMCN@PEG or Gd-DTPA (10 mg kg-1) at various time points, pseudo-color images (C), grayscale images (D). (E) Relative T_1 SNR of 4T1 tumor bearing BALB/c mice after i.v. injection of GMCN@PEG or Gd-DTPA (10 mg kg^{-1}) at various time points. (F, G) CT images of 4T1 tumor bearing BALB/c mice after i.v. injection of GMCN@PEG or Iohexol (10 mg kg^{-1}) at various time points, pseudo-color

images (F), grayscale images (G). (H) CT value of 4T1 tumor bearing BALB/c mice after i.v. injection of GMCN@PEG or Iohexol (10 mg kg^{-1}) at various time points.

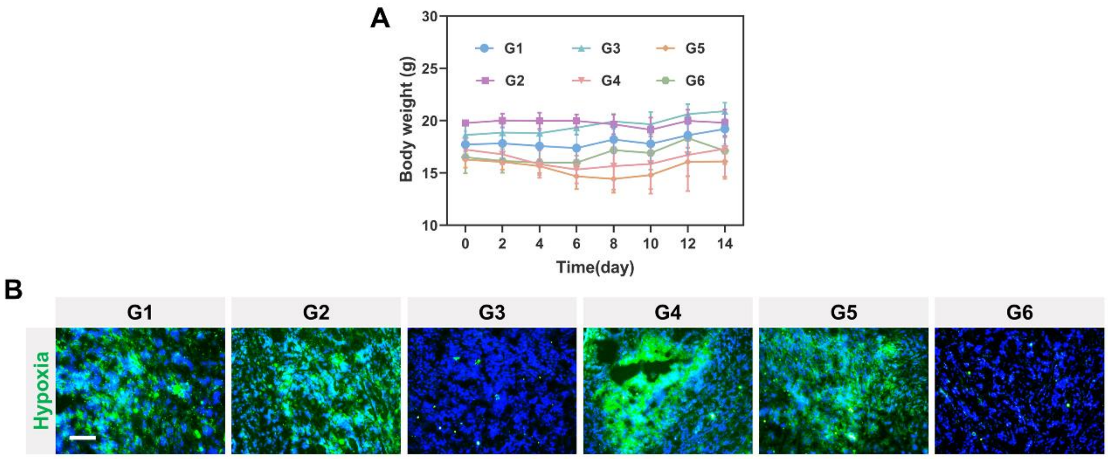

Figure S10. (A) Body weight curves of 4T1 tumor-bearing mice with various treatments in 4T1 Primary tumor mice model. (B) Representative images of tumor hypoxia across treatment groups in 4T1 primary tumor upon different treatments. Scale bar: 100 μm.

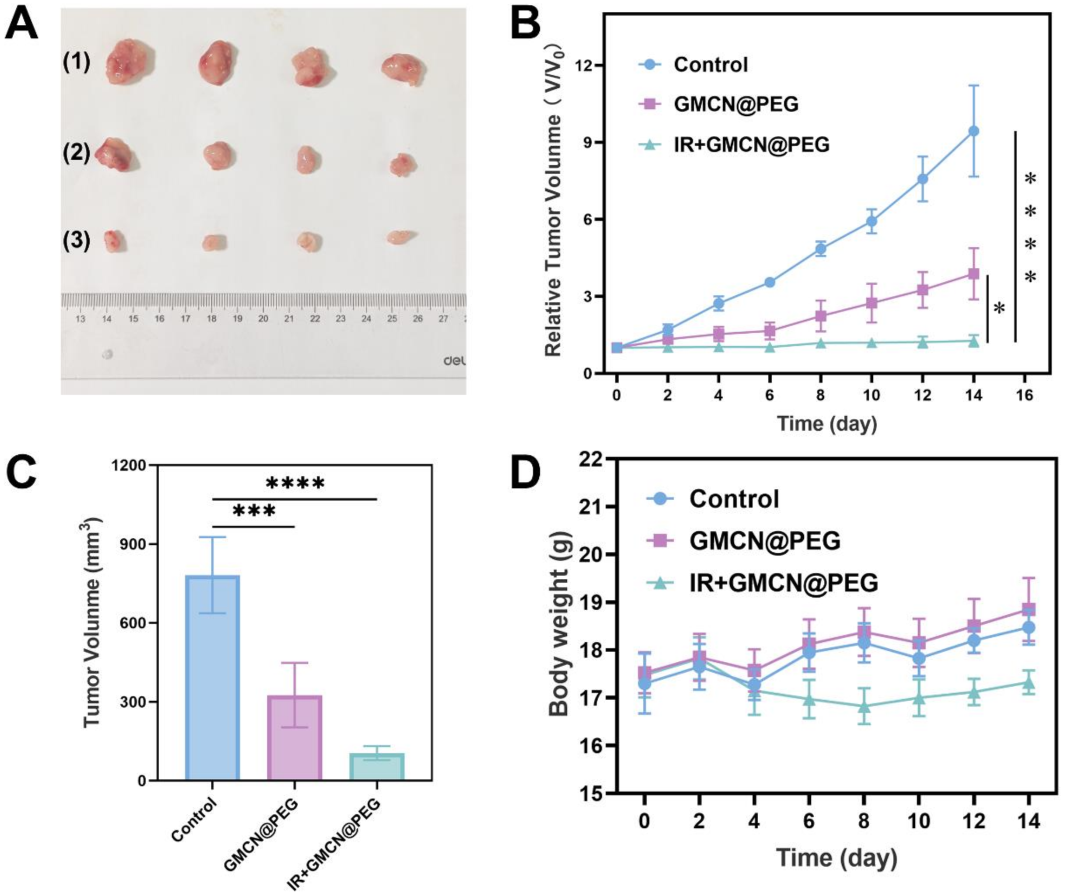

Figure S11. GMCN@PEG for radiotherapy enhancement in primary tumor-bearing mice via intravenous injection. (A) The image of the dissected primary tumors from each group on day 14. (1): Control; (2): GMCN@PEG; (3): IR+GMCN@PEG. (B) Average tumor growth curves of primary tumors after various treatments. (C) Average tumor volume of primary tumors from each group on day 14. (D) Body weight curves of 4T1 tumor-bearing mice with various treatments in 4T1 Primary tumor mice model. Statistical significance is indicated by asterisks: *$p < 0.05$, **$p < 0.01$, ***$p < 0.001$, ****$p < 0.0001$. Data are presented as means ± SD.

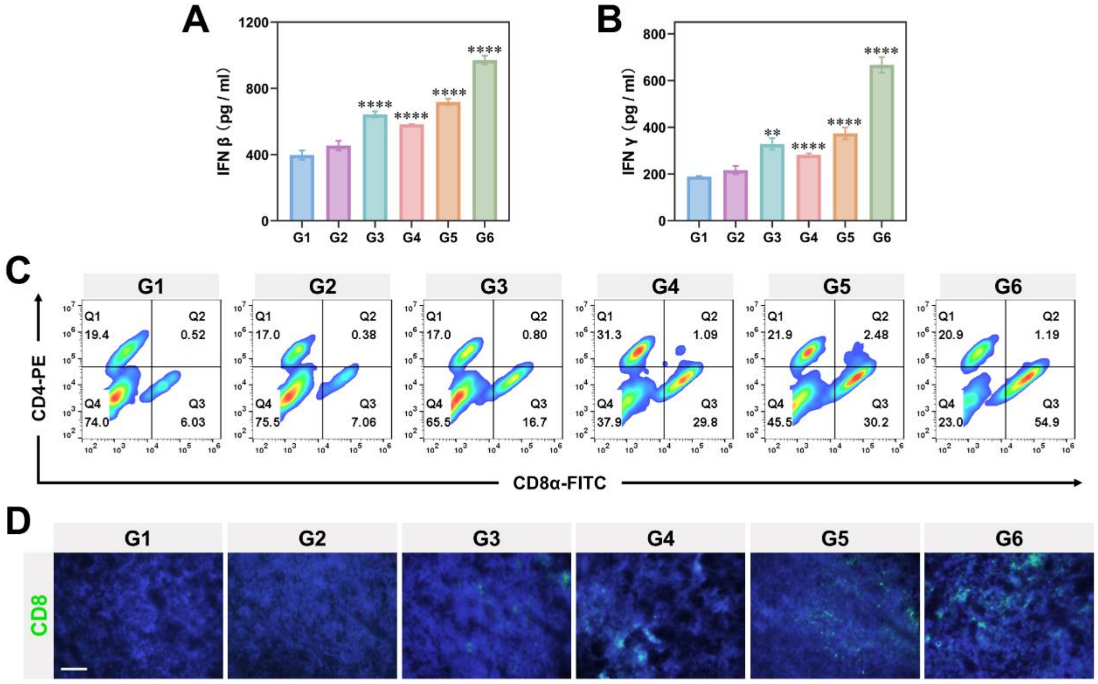

Figure S12. GMCN@PEG for radiotherapy-induced immune response enhancement in primary tumor-bearing mice. (A, B) Contents of the IFN-β (A) and IFN-γ (B) in primary tumors from each group after various treatments on day 14. (C) Flow cytometry analysis of $CD4^+$ and $CD8^+$ T cells (gated on $CD3^+$ T cells) in primary tumors after different treatments. (D) Immunofluorescence imaging of CD8 expression in 4T1 primary tumor upon different treatments. Scale bar: 100 μm. Statistical significance is indicated by asterisks: *$p < 0.05$, **$p < 0.01$, ***$p < 0.001$, ****$p < 0.0001$. Data are presented as means ± SD.

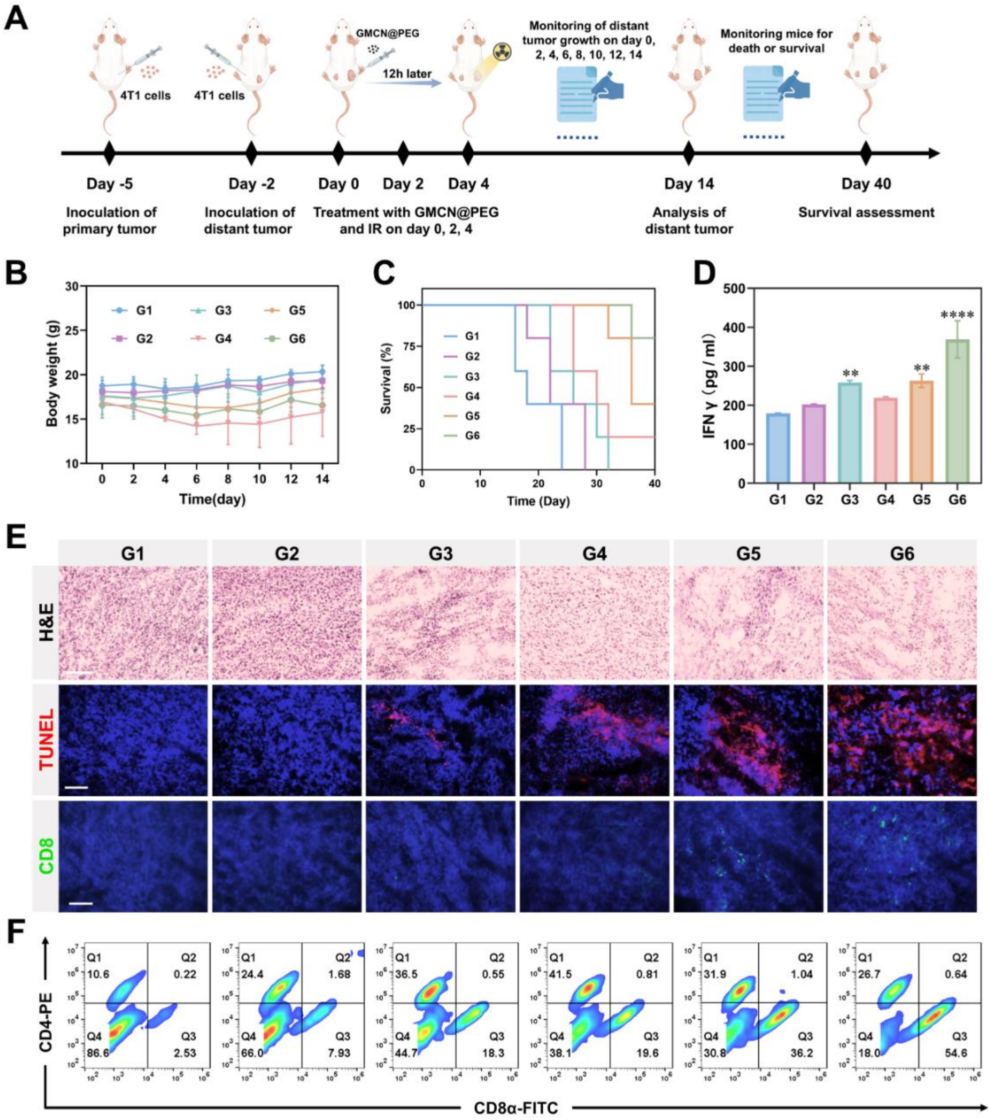

Figure S13. GMCN@PEG for radiotherapy enhancement in metastatic tumor-bearing mice. (A) Illustration of distant 4T1 tumor therapy *in vivo*. (B) Body weight curves of 4T1 tumor-bearing mice with various treatments in 4T1 metastatic tumor mice model. (C) Kaplan−Meier survival curves of 4T1 tumor-bearing mice with various treatments in 4T1 metastatic tumor mice model. (D) Contents of the IFN-γ in distant tumors from each group after various treatments. (E) Histological analysis with H&E, TUNEL and CD8 staining of primary tumor sections from each treatment group on day 14. (F) Flow cytometry analysis of $CD4^+$ and $CD8^+$ T cells (gated on $CD3^+$ T cells) in distant tumors after different treatments. Scale bar: 100 μm. Statistical significance is indicated by asterisks: *$p < 0.05$, **$p < 0.01$, ***$p < 0.001$, ****$p < 0.0001$. Data are presented as means ± SD.

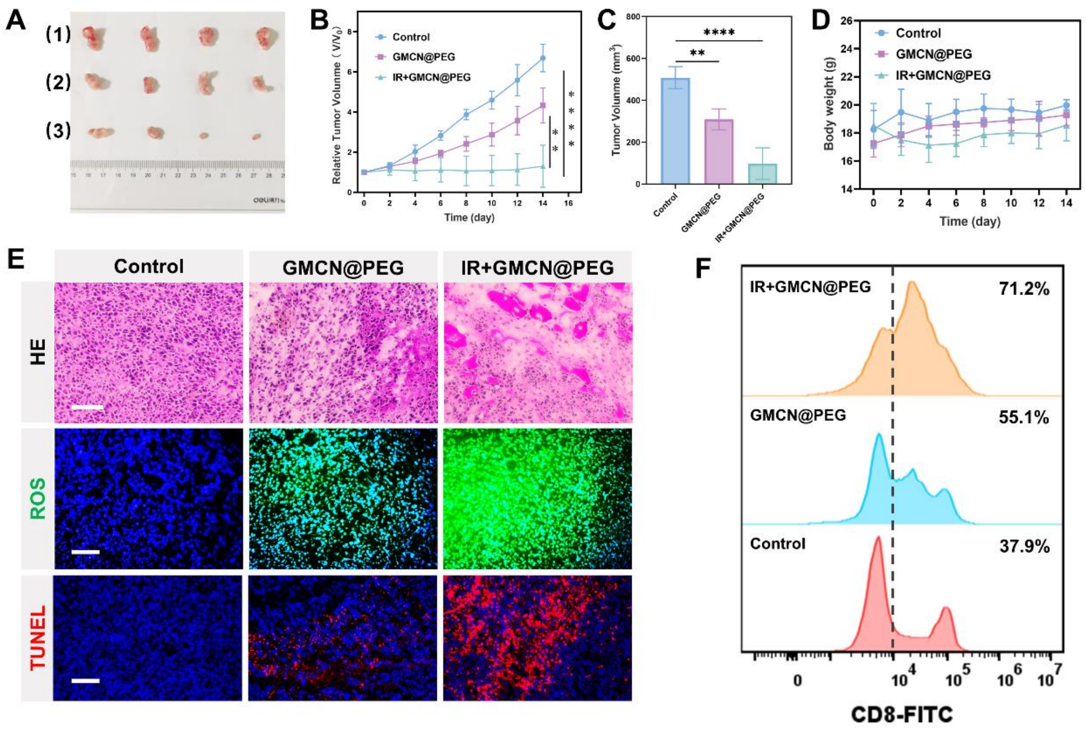

Figure S14. GMCN@PEG enhances radiotherapy in Hepatocellular Carcinoma tumor-bearing mice. (A) The image of the dissected tumors from each group on day 14. (1): Control; (2): GMCN@PEG; (3): IR+GMCN@PEG. (B) Average tumor growth curves of tumors after various treatments. (C) Average tumor volume of tumors from each group on day 14. (D) Body weight curves of tumor-bearing mice with various treatments. (E) Histological analysis with H&E, TUNEL and ROS staining of tumor sections from each treatment group on day 14. (F) Flow cytometry analysis of $CD8^+$ T cells (gated on $CD3^+$ T cells) in Hepatocellular Carcinoma after different treatments. Scale bar: 100 μm. Statistical significance is indicated by asterisks: *p < 0.05, **p < 0.01, ***p < 0.001, ****p < 0.0001. Data are presented as means ± SD.

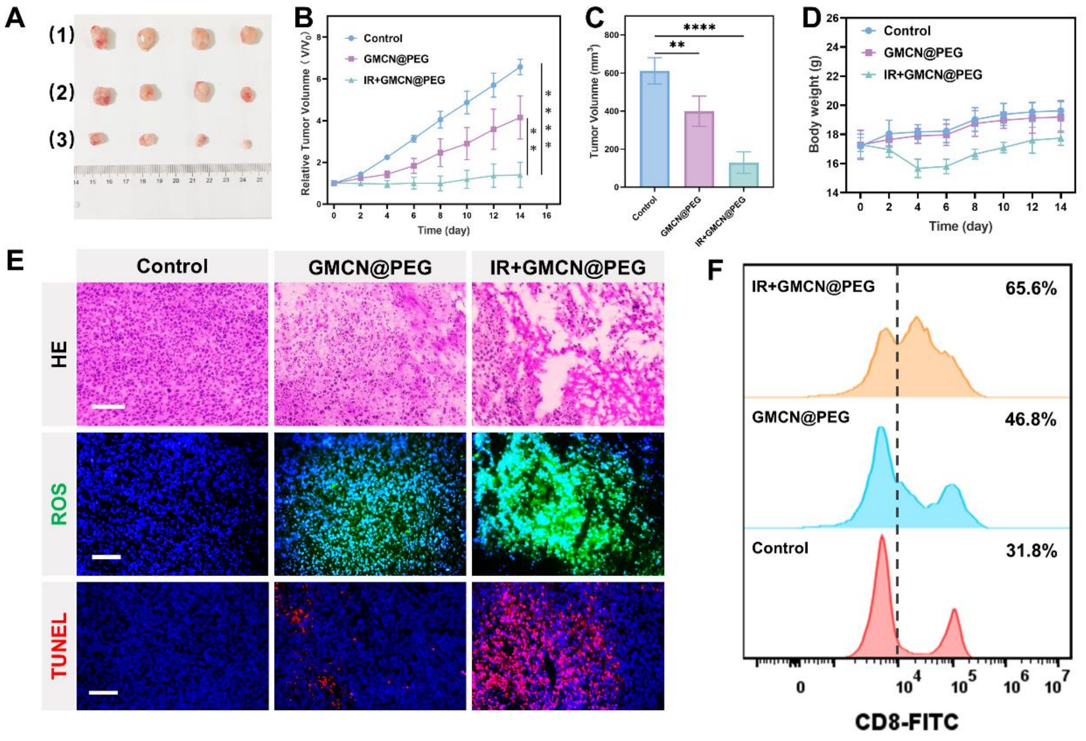

Figure S15. GMCN@PEG enhances radiotherapy in Colorectal Cancer tumor-bearing mice. (A) The image of the dissected tumors from each group on day 14. (1): Control; (2): GMCN@PEG; (3): IR+GMCN@PEG. (B) Average tumor growth curves of tumors after various treatments. (C) Average tumor volume of tumors from each group on day 14. (D) Body weight curves of tumor-bearing mice with various treatments. (E) Histological analysis with H&E, TUNEL and ROS staining of tumor sections from each treatment group on day 14. (F) Flow cytometry analysis of $CD8^+$ T cells (gated on $CD3^+$ T cells) in Colorectal Cancer after different treatments. Scale bar: 100 μm. Statistical significance is indicated by asterisks: *$p < 0.05$, **$p < 0.01$, ***$p < 0.001$, ****$p < 0.0001$. Data are presented as means ± SD.
